# Exploring diagnostic m6A regulators in endometriosis

**DOI:** 10.18632/aging.202163

**Published:** 2020-11-24

**Authors:** Li Jiang, Mengmeng Zhang, Jingni Wu, Sixue Wang, Xiang Yang, Mingyu Yi, Xinyue Zhang, Xiaoling Fang

**Affiliations:** 1 Department of Obstetrics and Gynecology, The Second Xiangya Hospital, Central South University, Changsha, Hunan, China

**Keywords:** endometriosis, N6-Methyladenosine, HNRNPA2B1, HNRNPC, immune system

## Abstract

Endometriosis is an estrogen-dependent inflammatory disorder, usually causing infertility, pelvic pain, and ovarian masses. This study intended to investigate the implication of N6-methyladenosine (m6A) regulators in endometriosis. We acquired 34 normal, 127 eutopic, and 46 ectopic, samples of endometrium from the Gene Expression Omnibus (GSE7305, GSE7307, GSE51981) database and the Array-express (E-MTAB-694) database. These samples were then used to profile the expression of 20 m6A regulators in endometriosis. The results indicated that most dysregulated (19/20) m6A regulators were significantly downregulated in eutopic *vs.* normal endometrium and also significantly downregulated in ectopic *vs.* eutopic endometrium. Several dysregulated m6A regulators were common to both contrast matrices: METTL3, YTHDF2, YTHDF3, HNRNPA2B1, HNRNPC, and FTO. Both HNRNPA2B1 and HNRNPC were associated with the severity of endometriosis in eutopic samples, and also exhibited diagnostic potential for endometriosis. HNRNPA2B1 and HNRNPC may influence immune pathways and the infiltration of immune cells in endometriosis. Abnormalities in the gene transcription factors network associated with endometriosis might affect the expression of HNRNPA2B1 and HNRNPC. In conclusion, we observed significant dysregulation of m6A regulators in endometriosis, and found that HNRNPA2B1 and HNRNPC might correlate with the immune response and serve as useful diagnostic biomarkers for endometriosis.

## INTRODUCTION

Endometriosis (EMs), defined by the implantation and growth of endometrial-like tissue outside of the uterine cavity, is an estrogen-dependent inflammatory disorder that afflicts approximately 10% of women worldwide [1, 2]. EMs is also considered to be a complex and heterogeneous condition because lesions can be found in a diverse range of anatomical locations, including the pelvic peritoneum, and various organs, such as the ovary, bladder, and rectum. EMs can also cause a range of non-specific symptoms, including chronic pelvic pain and infertility [1]; however, these non-specific symptoms can make EMs particularly challenging to diagnose, especially in the early stages of the disease. Moreover, we know very little about the specific pathogenesis of EMs. The classic theory, originally put forward by Sampson, stated that endometrial fragments pass *via* the fallopian tubes into the pelvic cavity and then undergo implantation and further growth [3]. However, Sampson’s theory cannot fully explain all aspects of EMs. Therefore, it is critical that we acquire a comprehensive understanding of the specific molecular mechanisms underlying EMs if we are to improve the diagnosis and treatment of EMs.

Previous research has suggested that epigenetic alterations might play a critical role in the development of EMs [4], including DNA methylation, histone acetylation, and microRNA dysregulation [5]. For instance, overexpression of the aromatase gene in endometriotic cells resulted in the sustained local production of estrogen, and was attributed to the hypomethylation of DNA in non-promoter regions [6]. However, RNA methylation, a reversible post-translational modification that epigenetically targets RNA molecules, has rarely been studied in EMs. N6-methyladenosine (m6A) methylation, the most common modification of RNAs, plays an important role in RNA splicing, translocation, stability, and translation [7]. The functional effects of m6A are achieved by a series of dynamic and interactive m6A regulators: (1) ‘writers’ (methyltransferases) such as KIAA1429 [8], METTL3, METTL14 [9], RBM15, RBM15B [10], WTAP [11], and ZC3H13 [12]; (2) ‘readers’ (RNA binding proteins), including the YTH family (YTHDF1/2/3 and YTHDC1/2) [13, 14], the heterogeneous nuclear ribonucleoproteins (hnRNPs), including HNRNPA2B1, HNRNPC, and RBMX (HNRNPG) [15, 16], the insulin-like growth factor 2 mRNA-binding protein family, including IGF2BP1/2/3 [17]; and (3) ‘erasers’ (demethylases), such as ALKBH5 [18] and FTO [19].

Emerging evidence implies that m6A methylation may play roles in tumor proliferation, differentiation, apoptosis, invasion, and metastasis [20]. Notably, the key features of EMs are known to include invasion, reduced apoptosis, and defective differentiation [1]. Moreover, m6A methylation has been shown to participate in the process of epithelial-mesenchymal transition (EMT) in cancer cells by promoting the decay of TGFβ1 mRNA and the translation of Snail mRNA [21, 22]. EMT was also reported to be activated in EMs when stimulated by hypoxia and estrogen via the TGFβ and Wnt pathways [23]. The functional roles of m6A in physiological and pathological immunity, such as T cell homeostasis and differentiation, anti-tumor and anti-viral immune responses, and lipopolysaccharide-induced inflammatory reactions have also been reported [24]. Since inflammation and abnormal immune responses are central processes in the development of EMs [1], it follows that m6A RNA methylation might also play a role in the pathogenesis of EMs.

The role of m6A, and its associated regulators, have already been studied in other diseases associated with the endometrium. For example, Liu et al. previously reported that the levels of m6A were reduced by approximately ~70% in patients with endometrial cancer, probably due to the mutation of METTL14 or the downregulation of METTL3. Furthermore, the reduced levels of m6A appeared to play an oncogenic role in patients with endometrial cancer by activating the AKT pathway [25]. In another study, Zhai et al. reported reduced levels of m6A in the endometrium and myometrium of women suffering from adenomyosis compared to endometrium from healthy candidates. This reduction in the level of m6A was induced by downregulation of the hub m6A regulator METTLE3 in the eutopic endometrium of patients with adenomyosis [26]. Therefore, it appears that m6A modifications might also be associated with endometrial abnormity in patients with EMs. However, the specific role of m6A, and its associated regulators, has yet to be studied in patients with EMs.

In the present study, we acquired data from the Gene Expression Omnibus (GEO) and Array-express databases, including 34 normal (NM), 127 eutopic (EU), and 46 ectopic (EC), samples of endometrium tissue. We used these samples to analyze the mRNA expression levels of 20 m6A regulators in EMs. We identified m6A regulators that showed shared differential expression when compared between EU and NM tissues, and between EC and EU tissues. And we attempted to correlate these shared regulators with clinical data to identify m6A regulators that could be used as diagnostic targets for EMs. We then evaluated the diagnostic value of these m6A candidates by receiver operating characteristic (ROC) analysis. Gene set enrichment analysis (GSEA), and single-sample gene set enrichment analysis (ssGSEA), were also used to annotate diagnostic m6A regulators, whose potential regulatory mechanisms were investigated by constructing a Gene-Transcription Factors (TFs) network. We also validated our results in an independent RNA-seq dataset GSE105764. To the best of our knowledge, our analysis represents the first endeavor to explore the potential implications of m6A regulators in EMs.

## RESULTS

### Data preprocessing

**Figure 1:**
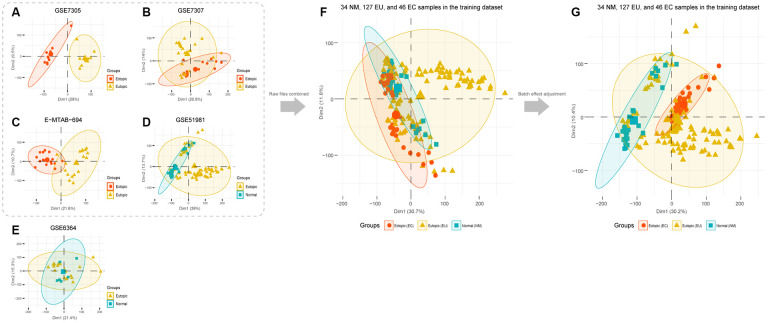
**PCA analysis of EMs microarray candidate datasets.** PCA analysis was performed in each microarray dataset: (**A**) GSE7305, (**B**) GSE7307, (**C**) E-MTAB-694, (**D**) GSE51981, and (**E**) GSE6364. (**F**) Datasets that showed good performance in the PCA analysis were selected to merge. (**G**) PCA analysis was performed after batch effect adjustment. EMs, endometriosis; PCA, the principal component analysis.

PCA analysis showed that EU samples could be easily distinguished from EC samples in the GSE7305, GSE7307, and E-MTAB-694 datasets. In the GSE51981 dataset, most NM samples could be distinguished from the EU samples (Figure 1A–1D). However, in the GSE6364 dataset, it was very difficult to differentiate EU and NM samples given that the central points of the PCA results almost overlapped (Figure 1E). To improve the quality of this analysis, we excluded the GSE6364 dataset from the subsequent combined analysis; this was due to non-satisfactory PCA performance.

Thus, we processed and merged raw CEL files for GSE7305, GSE7307, GSE51981, and E-MTAB-694. Then, we obtained a normalized matrix, that featured 34 normal (NM), 127 eutopic (EU), and 46 ectopic (EC) samples of the endometrium (Figure 1F). PCA analysis, carried out after adjustment for batch effects, indicated that the EC samples were obviously distinct from the NM samples while some EU samples were mixed with NM or EC samples. These findings suggested the innate heterogeneity of the EU samples (Figure 1G).

### The mRNA expression landscape of m6A regulators in EMs

**Figure 2:**
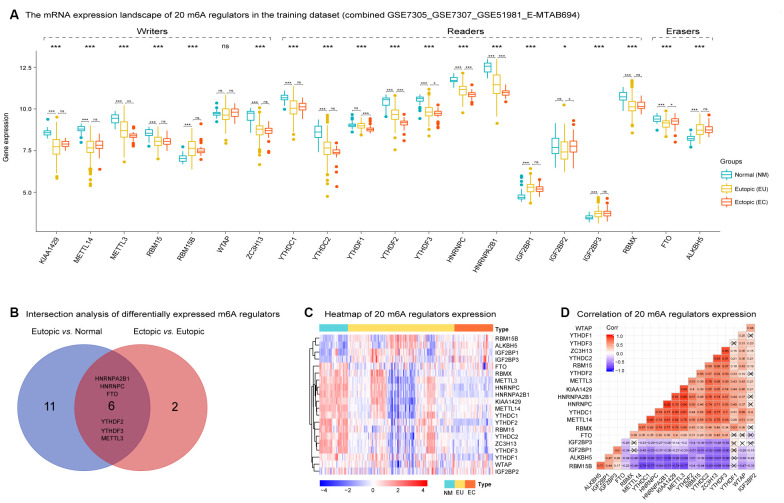
**The mRNA expression landscape of 20 m6A regulators in the training dataset in EMs.** (**A**) Most m6A regulators were dysregulated among NM, EU, and EC samples except for WATP ('Kruskal.test'). Several m6A regulators were differentially expressed in the EU vs. NM matrix and the EC vs. EU matrix, respectively ('LIMMA' R package). (**B**) Intersection analysis of differentially expressed m6A regulators between the EU vs. NM matrix and the EC vs. EU matrix. (**C**) The heatmap of 20 m6A regulators' expression among NM, EU, and EC samples. The heatmap was based on 'Euclidean' distance, and hierarchical clustering (clustering method = "complete" in R package 'pheatmap'); the clustering was performed on rows (genes) while not on columns (samples). (**D**) Spearman correlation analysis of 20 m6A regulators expression in EMs. EMs, endometriosis; NM, normal endometrium; EU, eutopic endometrium; EC, ectopic endometrium. NS - not significant; * p < 0.05; ** p < 0.01; *** p < 0.001.

The expression patterns of 20 m6A regulators in 34 NM, 127 EU, and 46 EC samples are shown in Figure 2A. Exception for WATP, most m6A regulators were dysregulated among three groups. When compared with NM samples, most m6A regulators were significantly downregulated in EU samples, including KIAA1429, METTL14, METTL3, RBM15, ZC3H13, YTHDC1, YTHDC2, YTHDF2, YTHDF3, HNRNPC, HNRNPA2B1, RBMX, and FTO. In contrast, RBM15B, IGF2BP1, IGF2BP3, and ALKBH5 were significantly upregulated in EU samples. However, when compared with EU samples, only a small number of m6A regulators showed significant changes in EC samples: METTL3, YTHDF1, YTHDF2, YTHDF3, HNRNPC, and HNRNPA2B1 were downregulated, while IGF2BP2 and FTO were upregulated (Supplementary Table 2). Moreover, METTL3, YTHDF2, YTHDF3, HNRNPC, HNRNPA2B1, and FTO, were identified as differentially expressed m6A regulators that were shared between the EU *vs.* NM matrix and the EC *vs*. EU matrix (Figure 2B).

The generation of a heatmap for the expression of the 20 m6A regulators revealed significant heterogeneity in expression in both the EU and EC samples (Figure 2C). Spearman correlation analysis of the 20 m6A regulators indicated that the highest positive correlation coefficient was detected between HNRNPC and HNRNPA2B1 (r = 0.92, p < 0.05) (Figure 2D).

### The relationship between selected m6A regulators and EMs

**Figure 3:**
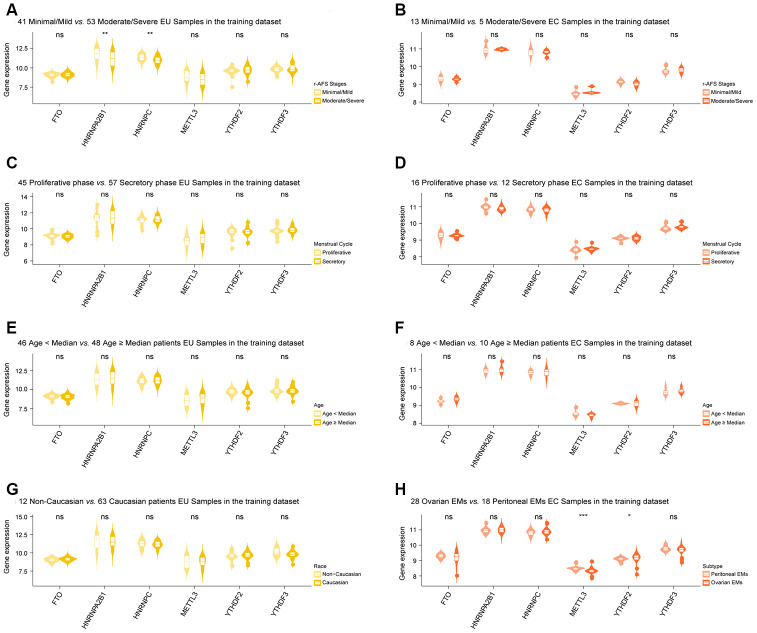
**Clinical correlation analysis of selected m6A regulators in EMs.** (**A**) HNRNPC and HNRNPA2B1 showed differential expression in different r-AFS stages in EU samples. However, no m6A regulators showed any significant changes across different r-AFS stages in EC samples (**B**), in different stages of the menstrual cycle, in different age groups for both EU and EC samples (**C**–**F**), and different races for the EU samples (**G**). Moreover, METTL3 and YTHDF2 were differentially expressed in different subtypes of EM in EC samples (**H**). ‘Wilcox.test’ was used for comparison between two groups. EMs, endometriosis; r-AFS, the revised American Fertility Society; NM, normal endometrium; EU, eutopic endometrium; EC, ectopic endometrium. NS - not significant; * p < 0.05; ** p < 0.01; *** p < 0.001.

Differentially expressed m6A regulators that were shared between the EU *vs.* NM matrix and the EC *vs.* EU matrix (METTL3, YTHDF2, YTHDF3, HNRNPA2B1, HNRNPC, and FTO) were chosen for clinical correlation analysis. This analysis indicated that HNRNPC and HNRNPA2B1 showed differential expression in different r-AFS stages in EU samples (Figure 3A). However, no m6A regulators showed any significant changes across different r-AFS stages in EC samples (Figure 3B), in different phases of the menstrual cycle, in different age groups for both EU and EC samples (Figure 3C–3F), and in different races for the EU samples (Figure 3G). Moreover, we found that METTL3 and YTHDF2 were differentially expressed in different subtypes of EMs in EC samples: METTL3 was downregulated in ovarian EC samples when compared to peritoneal EC samples, while YTHDF2 was upregulated in the ovarian EC samples (Figure 3H).

### The diagnostic value of HNRNPC and HNRNPA2B1 in EMS

**Figure 4:**
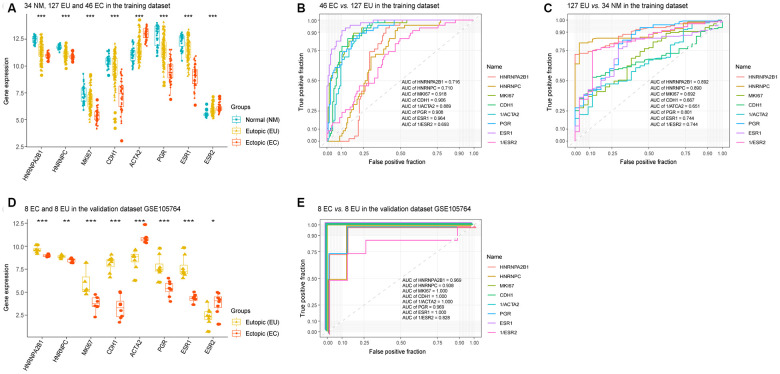
**The diagnostic value of HNRNPC and HNRNPA2B1 in EMs.** The expression levels of HNRNPA2B1, HNRNPC, MKI67, CDH1 (E-cadherin), ACTA2 (α-SMA), PGR, ESR1, and ESR2 in the training dataset (‘Kruskal.test’) (**A**) and validation dataset GSE105764 (‘DEseq2’) (**D**). ROC analysis of HNRNPA2B1, HNRNPC, MKI67, CDH1 (E-cadherin), ACTA2 (α-SMA), PGR, ESR1, and ESR2 in the EC vs. EU matrix (**B**) and the EU vs. NM matrix (**C**) in the training dataset, and the EC vs. EU matrix in validation dataset GSE105764 (**E**). EMs, endometriosis; NM, normal endometrium; EU, eutopic endometrium; EC, ectopic endometrium; ROC, receiver operating characteristic; AUC, areas under the curve. * p < 0.05; ** p < 0.01; *** p < 0.001.

Given that HNRNPA2B1 and HNRNPC were associated with the severity of EMs in EU samples, we selected these two molecules to evaluate their diagnostic value when compared to MKI67, CDH1 (E-cadherin), ACTA2 (α-SMA), PGR, ESR1, and ESR2, in EMs tissue samples. As shown in Figure 4A, HNRNPA2B1, HNRNPC, MKI67, CDH1 (E-cadherin), PGR, and ESR1, were all downregulated in the EU *vs.* NM matrix and the EC *vs.* EU matrix, while ACTA2 (α-SMA) and ESR2 were upregulated in the EU vs. NM and EC vs. EU matrices. Hence, 1/ACTA2 and 1/ESR2 were used to perform ROC analyses.

ROC analysis identified that HNRNPA2B1 (AUC = 0.892) and HNRNPC (AUC = 0.890) demonstrated higher diagnostic potential over MKI67 (AUC = 0.692), CDH1 (AUC = 0.667), 1/ACTA2 (AUC = 0.651), PGR (AUC = 0.801), ESR1 (AUC = 0.744) and 1/ESR2 (AUC = 0.744) in the EU *vs.* NM matrix (Figure 4C). HNRNPA2B1 (AUC = 0.716) and HNRNPC (AUC = 0.710) also exhibited relatively modest diagnostic value compared to MKI67 (AUC = 0.918), CDH1 (AUC = 0.906), 1/ACTA2 (AUC = 0.889), PGR (AUC = 0.908), ESR1 (AUC = 0.964) and 1/ESR2 (AUC = 0.693) in the EC vs. EU matrix (Figure 4B). Moreover, the scatter points showed a more concentrated distribution for HNRNPA2B1 and HNRNPC than for MKI67, CDH1, ACTA2, PGR and ESR1 (Figure 4A).

In addition, analysis of the validation dataset (GSE105764) also showed that HNRNPA2B1 and HNRNPC possessed diagnostic potential for discriminating between EC and EU samples, with an AUC of 0.969 and an AUC of 0.938 compared to MKI67 (AUC = 1.000), CDH1 (AUC = 1.000), 1/ACTA2 (AUC = 1.000), PGR (AUC = 0.969), ESR1 (AUC = 1.000) and 1/ESR2 (AUC = 0.828) (Figure 4D, 4E).

### Functional annotation of HNRNPA2B1 and HNRNPC in EMs

**Figure 5:**
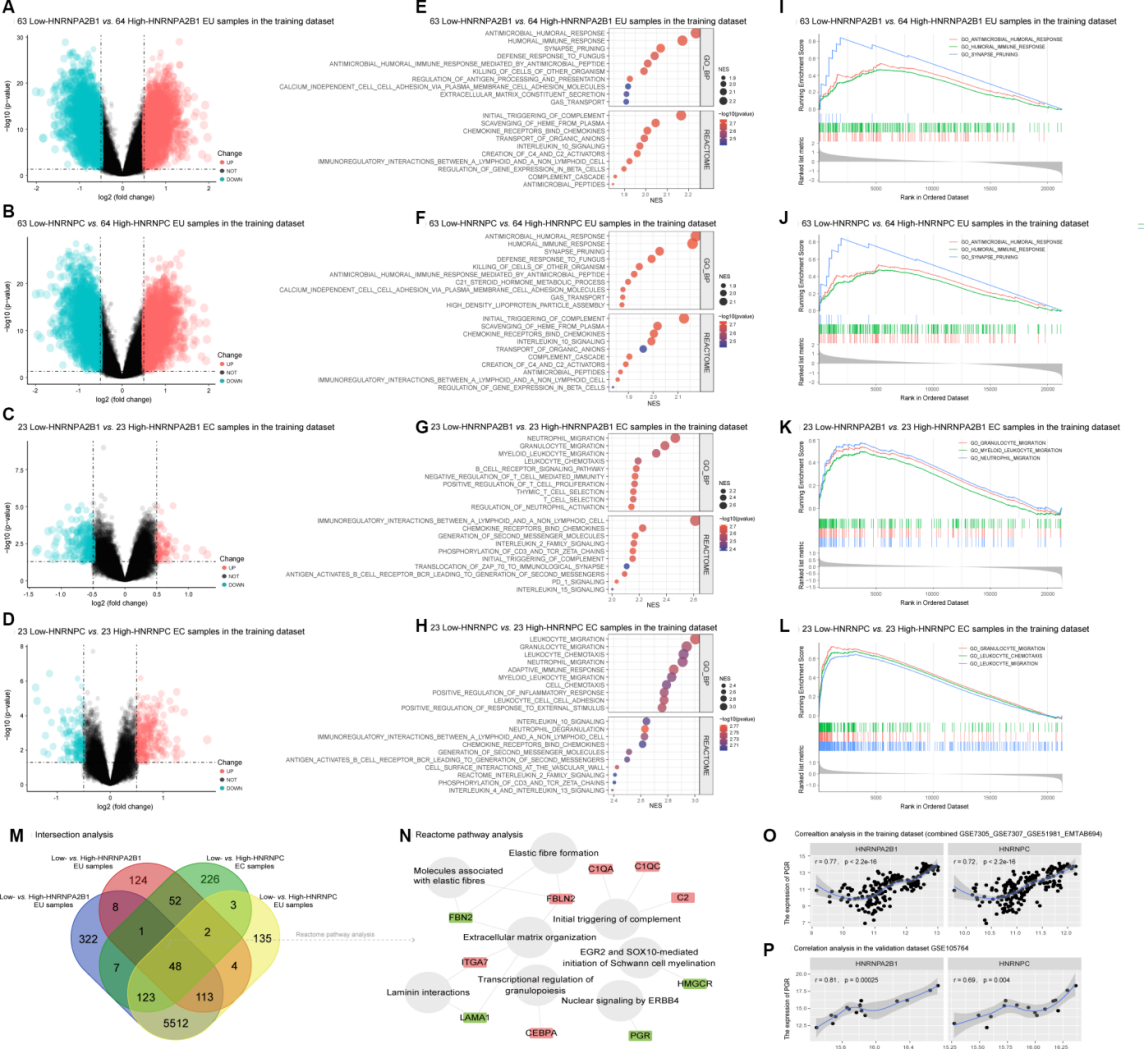
**Functional annotation of HNRNPA2B1 and HNRNPC in EMs.** (**A**–**D**) Differentially expressed genes (DEGs) between low-HNRNPA2B1 vs. high-HNRNPA2B1 EU samples, low-HNRNPC vs. high-HNRNPC EU samples, low-HNRNPA2B1 vs. high-HNRNPA2B1 EC samples, and low-HNRNPC vs. high-HNRNPC EC samples in the training dataset (Green dots, DEGs with log2FC < -0.5 and p < 0.05; Red dots, DEGs with log2FC > 0.5 and p < 0.05; Black dots, |log2FC| < 0.05 or p > 0.05). (**E**–**H**) The GSEA analysis of HNRNPA2B1 and HNRNPC in EMs. (**I**–**L**) Classical GSEA plots of the top 3 BP terms in each contrast matrix. (**M**) 48 shared DEGs (|log2FC| > 0.5 and p < 0.05) between low-HNRNPA2B1 vs. high-HNRNPA2B1 and low-HNRNPC vs. high-HNRNPC in EU and EC samples (**N**) The enriched Reactome pathways of 48 shared DEGs. (Grey circles, Reactome pathways; Red rectangles, up-regulated DEGs; Green rectangles, down-regulated DEGs). (**O**), (**P**) The correlation between PGR and HNRNPA2B1, HNRNPC in training and validation datasets. EMs, endometriosis; EU, eutopic endometrium; EC, ectopic endometrium; GSEA, the gene set enrichment analysis; BP, biological process; PGR, progesterone receptor.

To explore the putative function of HNRNPA2B1 and HNRNPC in EMs, we divided the EU and EC samples into high and low expression groups based on their median expression values. We then identified differentially expressed genes (DEGs) in 63 low-expression *vs.* 64 high-expression HNRNPA2B1 EU samples, 63 low-expression *vs.* 64 high-expression HNRNPC EU samples, 23 low-expression *vs.* 23 high-expression HNRNPA2B1 EC samples, and 23 low-expression *vs.* 23 high-expression HNRNPC EC samples. Volcano plots showed that the DEGs in EU samples were much more abundant than in the EC samples (Figure 5A–5D). GSEA analysis further indicated that HNRNPA2B1 and HNRNPC were involved in similar Biological Processes (BPs) and Reactome pathways, most of which were associated with immune and inflammatory responses (Figure 5E–5H). For example, in the EU samples, the enriched BP terms and Reactome pathways with the highest normalized enrichment score (NES) in the low-expression *vs.* high-expression HNRNPA2B1 samples, and the low-expression *vs.* high-expression HNRNPC samples, were both ‘antimicrobial humoral response’ and ‘initial triggering of complement’ (Figure 5E, 5F). The top 3 BP terms in each matrix were also presented as classical GSEA plots (Figure 5I–5L).

We also used the online Enrichr tool to carry out BP and KEGG analysis. This analysis also indicated that HNRNPA2B1 and HNRNPC were associated with immune and inflammatory pathways in both EU and EC samples. Moreover, these two molecules were related to RNA metabolic process, RNA transportation, RNA splicing, and cell cycle pathways (Supplementary Figure 1). Furthermore, 48 shared DEGs were obtained from screened DEGs (|log2FC| > 0.5 and p < 0.05) between low-expression *vs.* high-expression HNRNPA2B1 and low-expression *vs.* high-expression HNRNPC in respective EU and EC samples (Figure 5M). Most of these were enriched in Reactome pathways related to tissue remolding, such as ‘molecules associated with elastic fibers’, ‘elastic fiber formation’, ‘extracellular matrix organization’, and immune response such as ‘initial triggering of complement’ (Figure 5N).

Notably, PGR was one of the 48 shared DEGs and was downregulated in the low-expression HNRNPA2B1 and low-expression HNRNPC samples compared to high-expression HNRNPA2B1 and high-expression HNRNPC samples (Figure 5N). PGR was also positively correlated with the expression of HNRNPA2B1 (r = 0.77, p < 0.05) and HNRNPC (r = 0.72, p < 0.05) in both the training and validation datasets (Figure 5O, 5P).

### The association of HNRNPA2B1 and HNRNPC with infiltrating immune cells in EMs

**Figure 6:**
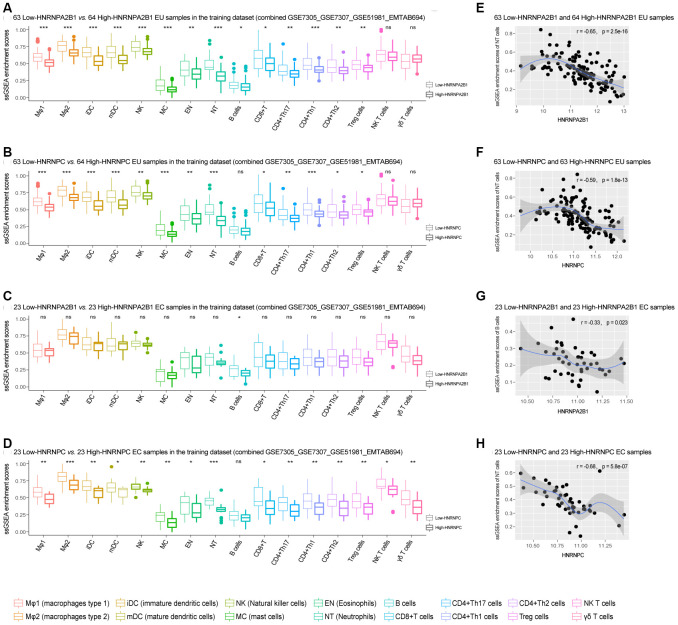
**The association of HNRNPA2B1 and HNRNPC with infiltrating immune cells in EMs.** (**A**–**D**) Differentially expressed ssGSEA scores of 16 kinds of immune cells between low-HNRNPA2B1 vs. high -HNRNPA2B1 and low-HNRNPC vs. high-HNRNPC, respectively, in the EU and EC samples (‘Wilcox.test’). (**E**–**H**) The association of HNRNPA2B1 and HNRNPC with one representative class of immune cells in the EU and EC samples. EMs, endometriosis; EU, eutopic endometrium; EC, ectopic endometrium; ssGSEA, single sample gene set enrichment analysis; NT, Neutrophils. NS - not significant, * p < 0.05; ** p < 0.01, *** p < 0.001.

ssGSEA analysis indicated that compared with the high-expression HNRNPA2B1 or HNRNPC EU samples, multiple types of immune cells showed significant elevation in the low-expression HNRNPA2B1 or HNRNPC EU samples (Figure 6A, 6B). Similarly, the infiltration scores for immune cells were generally higher in the low-expression HNRNPA2B1 or HNRNPC EC samples compared to the high-expression HNRNPA2B1 or HNRNPC EC samples, although only B cells showed a significant elevation in the low-expression *vs.* high-expression HNRNPA2B1 EC samples (Figure 6C, 6D). Moreover, the enrichment of immune cells was negatively correlated with the expression of HNRNPA2B1 and HNRNPC in both EU and EC samples (Figure 6E–6H, Supplementary Figure 2).

Furthermore, the MCP-counter method also showed that immune cells were more abundant in the low-expression HNRNPA2B1 or HNRNPC EU and EC samples compared to the high-expression HNRNPA2B1 or HNRNPC EU and EC samples (Supplementary Figure 3). The negative correlation was also observed between MCP-counter scores and the expression of HNRNPA2B1 and HNRNPC in both EU and EC samples (Supplementary Figure 4).

### The potential regulatory mechanisms underlying the role of HNRNPA2B1 and HNRNPC in EMs

**Figure 7:**
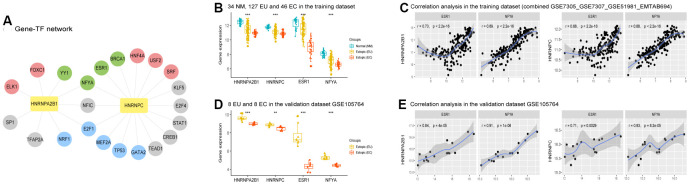
**Predicted transcription factors (TFs) of HNRNPA2B1 and HNRNPC in EMs.** (**A**) Predicted Gene-TF network of HNRNPA2B1 and HNRNPC in the training dataset. (Grey circles, unshared differentially expressed TFs between the EU vs. NM matrix and the EC vs. EU matrix; Red circles, TFs upregulated both in the EU vs. NM matrix and EC vs. EU matrix (p < 0.05); Green circles, TFs downregulated both in the EU vs. NM matrix and EC vs. EU matrix (p < 0.05); Blue circles, TFs with contrary expression trend in the EU vs. NM matrix and EC vs. EU matrix (p < 0.05). (**B**, **D**) The expression of ESR1 and NFYA in training (‘Kruskal.test’) and validation (‘DEseq2’ package) datasets in EMs. (**C**, **E**) The expression of HNRNPA2B1 and HNRNPC positively correlated with ESR1 and NFYA in training and validation datasets in EMs. EMs, endometriosis; NM, normal endometrium; EU, eutopic endometrium; EC, ectopic endometrium. * p < 0.05; ** p < 0.01; *** p < 0.001.

NetworkAnalyst version 3.0 predicted 9 transcription factors (TFs) for HNRNPA2B1 and 16 for HNRNPC. These predicted TFs were then used to construct a gene-TFs network (Figure 7A). Several of the predicted TFs were differentially expressed in the training dataset, among which SRF, ELK1, USF2, FOXC1, HNF4A were upregulated, and BRCA1, ESR1, YY1, NFYA were downregulated, both in the EU *vs.* NM matrix and the EC *vs.* EU matrix. The analysis also showed that TP53, E2F1, GATA2, NRF1, and MEF2A, presented with contrasting trends for these two matrices; for instance, TP53 was upregulated in the EU *vs*. NM matrix but was downregulated in the EC *vs.* EU matrix (Figure 7A, 7B and Supplementary Table 3).

Both HNRNPA2B1 and HNRNPC were significantly and positively correlated with the expression levels of NFYA (one of the common predicted TFs for HNRNPA2B1 and HNRNPC), and ESR1 (one of the putative TFs for HNRNPC), both of which were downregulated in the EU *vs.* NM and EC *vs.* EU matrices (Figure 7B, 7C). Similar results were observed in the GSE105764 validation dataset (Figure 7D, 7E and Supplementary Table 4).

## DISCUSSION

Endometriosis (EMs) is a common gynecological disease with heterogeneous manifestations and enigmatic pathogenesis [1–4]. The epigenetic role of N6-methyladenosine (m6A) methylation in various biological processes involving RNA has been reported in numerous forms of cancer [20] and adenomyosis [26] but not in EMs. This study represented the first attempt to investigate the implication of m6A regulators in EMs.

In this study, we analyzed the expression of 20 m6A regulators in 34 normal (NM), 127 eutopic (EU), and 46 ectopic (EC) samples of endometrium tissue that were merged from public microarray datasets of EMs. Our results showed that most differentially expressed m6A regulators were significantly downregulated in EU samples compared to NM samples and also downregulated in EC samples compared to EU samples. These findings probably indicate reduced m6A methylation levels in EMs; a similar phenomenon has been observed in endometrial cancer [25] and adenomyosis [26]. The reduced levels of m6A methylation play an oncogenic role in endometrial cancer, thus promoting the proliferation and tumorigenicity of endometrial cancer cells by activating AKT signaling [25]. The activation of AKT signaling has been observed in EMs ectopic tissue, endometriotic stromal cells, and eutopic endometrial stromal cells, but not in EMs-free women, thus supporting the proliferation and survival of ectopic endometrial tissues [27]. Moreover, an AKT inhibitor was shown to reduce the number of ectopic lesions in mice models of EMs [27]. Hence, we hypothesized that the relationship between reduced levels of m6A methylation and the AKT pathway in endometrial cancer might also be involved in the pathobiological mechanisms underlying EMs.

Moreover, we found that six m6A genes (METTL3, YTHDF2, YTHDF3, HNRNPC, HNRNPA2B1, and FTO) were differentially expressed and shared between the EU *vs.* NM matrix and the EC *vs.* EU matrix. METTL3 is the core methyltransferase of the m6A ‘writer’ complex and catalyzes the m6A methylation process [9]; the downregulation of this gene led to the reduction of m6A methylation in endometrial cancer [25], while its upregulation and the oncogenic role has been observed in many other forms of cancer, including ovarian [28] and breast cancers [29]. In our present study, METTL3 was downregulated in the EU *vs.* NM matrix and the EC *vs.* EU matrix and was also downregulated in ovarian EC samples compared to peritoneal EC samples. These data suggested that the METTL3 gene was tissue-specific, although further studies are needed to identify whether this gene relates to the prevention of malignant transformation in ovarian EMs. In endometrial cancers, the knockdown of YTHDF2, a ‘reader’ protein that acts to promote the decay of its target mRNAs, can impede the decay of mTORC2, thus activating the AKT pathway [25], which is also known to be triggered in EMs [27]. YTHDF3 is also thought to act with YTHDF2 during the decay process of methylated mRNA [30]. It is possible that estrogen could induce the expression of FTO (the fat mass and obesity-associated gene), in a manner that is depending on ESR1 (ESRα), promoting the proliferation of endometrial cancer cells [31]. However, the ‘protagonist’ in EMs is thought to be ESR2 (ESRβ); the preferential expression of this gene would reduce the expression level of ESR1 in endometriotic stromal cells [32]. Hence, we speculated that the under-expression of FTO in EU and EC samples, compared to NM samples, might be related to the aberrantly high ESR2/ESR1 ratio in EMs.

Furthermore, HNRNPA2B1 and HNRNPC, two members of the hnRNPs family that play roles in various RNA-related processes, such as pre-mRNA splicing [33], were also found to be correlated with the severity of EU samples, regardless of different ages, races, and menstrual cycle stages in patients with EMs. However, this correlation was not observed in EC samples, probably due to the limited sample size or the complex peritoneal microenvironment. These two molecules also exhibited diagnostic potential, particularly with regards to discriminating between EU and NM samples, when compared to MKI67, CDH1 (E-cadherin), ACTA2 (α-SMA), PGR, ESR1, and ESR2 in EMs tissue samples. Interestingly, both HNRNPA2B1 and HNRNPC were positively correlated with ESR1 and PGR; the downregulation of these genes is known to be the driver of estrogen-induced inflammation and progesterone resistance in EMs [32, 34]. Moreover, the common transcription factor for HNRNPA2B1 and HNRNPC, NFYA, has also been reported to be downregulated during the proliferative phase in EC samples compared to EU samples at the mRNA level (as determined by real-time PCR), but was upregulated in EU samples when compared to NM samples [35]. These findings are somewhat different from our present results, probably due to other detection methods and single-phase samples.

Although little is known of the role of HNRNPC in EMs, the protein levels of HNRNPA2B1 have been reported to be under-expressed in both EU and EC samples compared to NM samples, as determined by immunohistochemistry and western blot assays; although there was no significant change between the EU and EC samples, probably due to the limited sample size [36]. Moreover, HNRNPA2B1 was found to be differentially expressed in stage III/IV and stage II/I eutopic endometrium from patients with EMs [37]. The functional annotation of HNRNPA2B1 and HNRNPC indicated that both of these genes might be involved with the dysregulated immune response in EMs. For instance, low expression levels of HNRNPA2B1 and HNRNPC were associated with the high enrichment of NK cells in EU samples. NK cells are a critical member of the innate immune system; an impairment in the quantity, maturation, and cytotoxicity, of NK cells, will contribute to the aberrant endometrial development, poor implantation, and/or pregnancy outcomes in patients with EMs [38]. The lower expression levels of HNRNPA2B1 and HNRNPC were also associated with higher levels of enrichment for several other innate and adaptive immune cells in EU and EC samples; these dysregulated immune cells play a well-established role in the defective immunity of patients with EMs [38]. Notably, the involvement of HNRNPA2B1 and HNRNPC in dysregulated immunity has already been investigated by DNA viral infection [39], autoimmune endocrine disorders [40], and cancer diseases [41]. It is noteworthy that our results indicate that EMs patients have low expression levels of HNRNPA2B1 and HNRNPC, even though most other tumors are associated with high expression levels of HNRNPA2B1 and HNRNPC [20], except for in cases with kidney renal clear cell carcinoma [42]. Collectively, these findings suggest that these two genes are involved in different mechanisms in different tissues and diseases; further research is therefore required.

Meanwhile, our analysis has some limitations that need to be considered. Firstly, differences in expression levels were not stratified according to menstrual cycle phases (proliferative phase and secretory phase) or each stage of the r-AFS; mRNA profiles may undergo changes at different points of the menstrual cycle or in different r-AFS stages. Secondly, the diagnostic value of HNRNPA2B1 and HNRNPC was only validated in the GSE105674 dataset; this dataset only had a small sample size and only included EU and EC samples, not NM samples. Thirdly, the diagnostic value of HNRNPA2B1 and HNRNPC was only examined in tissue samples; further validation is required using samples of blood. Fourthly, the analysis was only performed at the mRNA level in EMs tissue samples; further validation is required at the protein level. Finally, the regulatory mechanisms associated with m6A RNA methylation in EMs still need to be investigated by a combination of *in vitro* and *in vivo* experiments.

In conclusion, our analyses identified significant dysregulation of m6A regulators in endometriosis. We also found that most of the dysregulated m6A regulators were significantly downregulated in eutopic samples when compared with normal endometrium, and were also downregulated in ectopic samples when compared with eutopic endometrium; this might indicate reduced levels of m6A methylation in endometriosis. Furthermore, HNRNPA2B1 and HNRNPC were both differentially expressed m6A regulators and common to the eutopic *vs.* normal endometrium and ectopic *vs.* eutopic endometrium. It is likely that these changes in the expression of HNRNPA2B1 and HNRNPC are associated with the abnormal immune response and that these factors may serve as efficient m6A-related diagnostic biomarkers in endometriosis.

## MATERIALS AND METHODS

### Data resources

**Figure 8:**
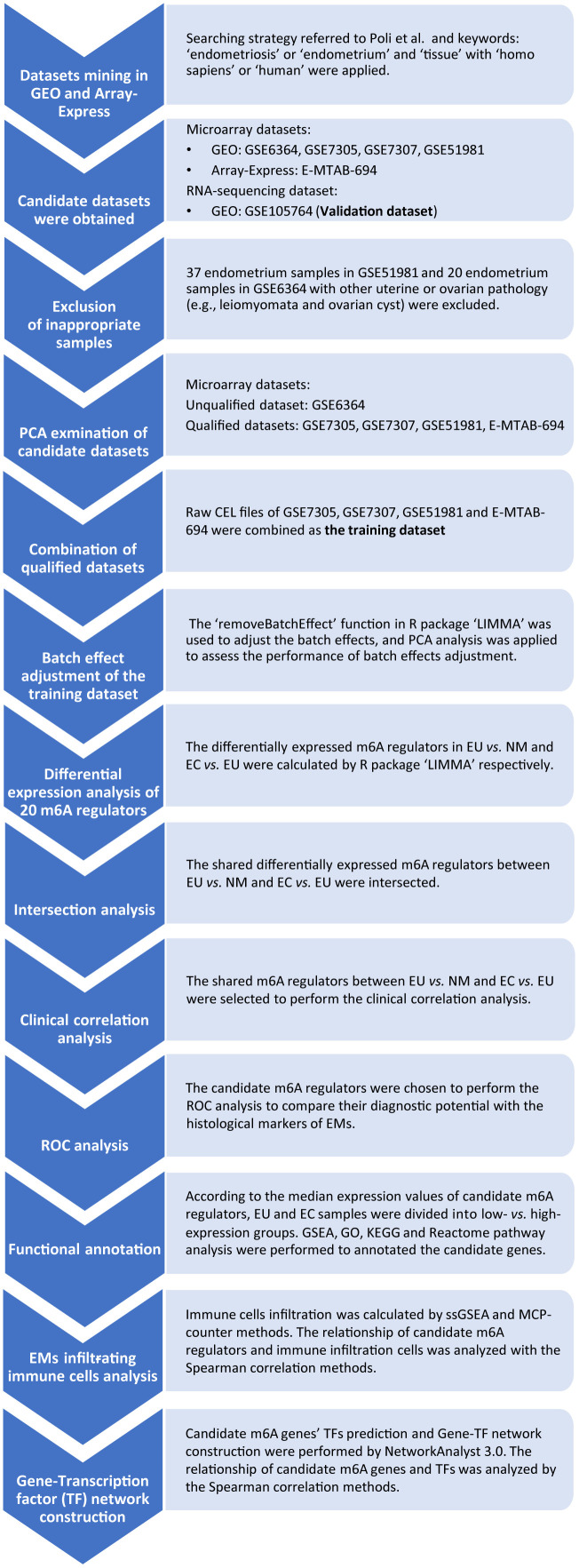
**The flowchart of m6A regulators’ analysis in EMs.** EMs, endometriosis; NM, normal endometrium; EU, eutopic endometrium; EC, ectopic endometrium; GEO, Gene Expression Omnibus; PCA, the principal component analysis; ROC, the receiver operating characteristic analysis; GSEA, gene set enrichment analysis; ssGSEA, single sample gene set enrichment analysis; GO, Gene Ontology; KEGG, Kyoto Encyclopedia of Genes and Genomes; MCP-counter, microenvironment cell populations counter; TF, transcription factor.

The analysis flowchart is shown in Figure 8. The GEO (https://www.ncbi.nlm.nih.gov/geo/) and Array-Express (https://www.ebi.ac.uk/arrayexpress/) databases were used to mine datasets associated with EMs. The search strategy was previously described by Poli et al. [43] and used the following keywords for the initial screen: ‘endometriosis’ or ‘endometrium’ and ‘tissue’ with ‘homo sapiens’ or ‘human’. Subsequently, we used the ‘platform GPL570’ (Affymetrix Human Genome U133 Plus 2.0 Array) as an additional filter condition to reduce the ‘platform effect’.

Our searches identified five candidate microarray datasets: GSE7305 [44], GSE7307, GSE51981 [45], GSE6364 [46], and E-MTAB-694 [47]. Further screening led to the exclusion of 37 endometrium samples in GSE51981 and 20 endometrium samples in GSE6364 due to the fact that these samples were associated with other forms of uterine or ovarian pathology (e.g., leiomyomata, adenomyosis, and ovarian cyst). Thus, 10 EU and 10 EC samples from GSE7305; 23 EU and 18 EC samples from GSE7307; 77 EU and 34 NM samples from GSE51981, 21 EU and 7 NM samples from GSE6364, and 17 EU and 18 EC samples from E-MTAB-694 were reserved. Most of the RNA-seq datasets associated with EMs usually involved small sample sizes and did not usually include NM, EU, and EC samples simultaneously. Therefore, we selected an independent RNA-seq dataset, GSE105764 [48], from the GEO database as a validation cohort; this dataset included 8 paired EU and EC tissue samples and was based on the GPL20301 platform (Illumina HiSeq 4000) (Table 1).

**Table 1:** Basic information of the retrieved datasets of Ems.

**Datasets**	**Accession**	**Platform**	**No. of probes**	**NM samples**	**EU samples**	**EC samples**	**References**
Microarray	GSE7305	GPL570 [HG-U133_Plus_2]	54675	-	10	10	Hever et al., 2007
	GSE7307	GPL570 [HG-U133_Plus_2]	54675	-	23	18	Unpublished
	GSE6364	GPL570 [HG-U133_Plus_2]	54675	7	21	-	Burney et al.,2007
	E-MTAB-694	GPL570 [HG-U133_Plus_2]	54675	-	17	18	Sohler et al., 2013
	GSE51981	GPL570 [HG-U133_Plus_2]	54675	34	77	-	Tamaresis et al., 2014
RNA-seq	GSE105764	GPL20301 [Illumina HiSeq 4000]	-	-	8	8	Zhao et al., 2018

Notes. EMs, endometriosis; NM, normal endometrium; EU, eutopic endometrium; EC, ectopic endometrium (lesions).

Clinical information associated with these datasets was retrieved from published articles (Supplementary Table 1). Clinical information was not available for GSE7307 as this data analysis had not been published. Ethical approval was not necessary as our analysis was based on public data resources, and did not involve direct experiments on humans or animals.

### Data preprocessing

First, raw CEL files were downloaded from the GEO and Array-Express databases. Second, we adjusted data for background correction and normalization using the robust multi-array average (RMA) method in the R package ‘Oligo’ [49]. Finally, probes were annotated according to the GPL570 annotation file; for this, we used the official Affymetrix website (http://www.affymetrix.com). Before combining data, we then performed principal component analysis (PCA) on each microarray dataset to examine dataset quality. Datasets that showed good performance in the PCA were selected for combing as the training dataset; datasets that showed poor performance in the PCA were excluded. The raw CEL files of the qualifying datasets were then combined in the R package to create a merged and normalized matrix containing NM, EU, and EC samples. We also used the ‘removeBatchEffect’ function in the R package ‘LIMMA’ [50] to adjust batch effects, and then carried out PCA analysis to evaluate the performance of batch effect adjustment.

### The mRNA expression landscape of m6A regulators in EMs

Following pretreatment, the expression levels of 20 m6A regulators were evaluated in samples of NM, EU, and EC tissues by the ‘Kruskal.test’ in the R Package. We also used the R package ‘LIMMA’ to identify differentially expressed m6A regulators in two contrasting matrices: EU *vs*. NM and EC *vs.* EU [50] with a threshold of p < 0.05. The differentially expressed m6A regulators that were shared between the EU *vs.* NM matrix and the EC *vs.* EU matrix were then screened out by intersection analysis. The expression of these 20 m6A regulators was then demonstrated in a heatmap using ‘pheatmap’ R package; this was based on Euclidean distance and hierarchical clustering [51]. We also evaluated the correlations between these m6A regulators in EMs using Spearman’s correlation analysis with a statistical threshold of p < 0.05.

### The relationships between the selected m6A regulators and EMs

The differentially expressed m6A regulators that were shared between the EU *vs.* NM matrix and the EC *vs.* EU matrix were then used for clinical correlation analysis. Clinical information is provided in Supplementary Table 1. Using this information, we investigated the associations between the selected m6A regulators and the key clinical features of patients with EMs, including the revised American Fertility Society (r-AFS) stages, menstrual cycle phases, age, races, and disease subtypes (ovarian EMs or peritoneal EMs). This analysis was carried out for both EU and EC samples and involved the ‘Wilcox.test’ in the R package with a statistical threshold of p < 0.05.

We did not analyze samples if clinical information was missing. We found that information relating to the race was missing from the EC samples; consequently, we did not perform race analysis on the EC samples. Furthermore, subtype correlation analysis was not performed for the EU samples as many of these EU samples were associated with multiple diagnoses simultaneously; for instance, some patients with ovarian EMs also had peritoneal EMs.

### Diagnostic value of selected m6A regulators in EMs

Next, we selected the m6A regulators that were significantly associated with the severity of EMs to perform receiver operating characteristic (ROC) analysis. This allowed us to compare the diagnostic potential of these m6A regulators with certain histological mRNA markers of EMs, including MKI67, CDH1 (E-cadherin), ACTA2 (α-SMA), PGR, ESR1, and ESR2. MKI67 is a classic marker of cell proliferation; proliferative activity is known to be limited in ectopic lesions due to the reduced ratio of epithelial/stromal cells [1, 52]. The process of epithelial-mesenchymal transition (EMT) is often marked by the loss of certain epithelial markers, including CDH1 (E-cadherin), and the acquisition of certain mesenchymal markers, such as ACTA2 (α-SMA). These processes, along with fibroblast-to-myofibroblast trans-differentiation (FMT), are thought to contribute to the increased production of collagen and smooth muscle metaplasia (SMM), thus resulting in fibrosis in EMs [53, 54]. Moreover, previous research has shown that the downregulation of ESR1 and PGR, and the upregulation of ESR2, play a central role in the estrogen-driven inflammation and progesterone resistance in EMs [55]. The results derived from our analyses were validated in the GSE105764 dataset. A p-value < 0.05 was regarded as statistically significant.

### Functional annotation of diagnostic m6A regulators in EMs

To explore the putative function of diagnostic m6A regulators in EMs, we divided the EU and EC samples into two groups according to their median expression values: a low expression group and a high expression group. We then used the ‘LIMMA’ R package to identify differentially expressed genes (DEGs) between these two groups. Next, we used the ‘Clusterprofiler’ R package to perform gene set enrichment analysis (GSEA) analysis [56]. And we used two gene sets to act as reference gene sets: ‘c5.bp.v7.0.symbols.gmt’ for Biological Process (BP) terms for Gene Ontology (GO) analysis, and ‘c2.cp.reactome.v7.0.symbols.gmt’ for Reactome Pathway analysis. These gene sets were downloaded from the Molecular Signature Database (MSigDB, https://www.gsea-msigdb.org/gsea/msigdb/index.jsp). P-values < 0.01 and adjusted p-values < 0.05 were considered to be statistically significant.

Then, the DEGs that were identified between the low expression and high expression groups, which showed a |log2FC| > 1 and p < 0.05 in the EU samples, and DEGs with a |log2FC| > 0.5 and a p < 0.05 in the EC samples, were used for BP and Kyoto Encyclopedia of Genes and Genomes (KEGG) pathway analysis. For this analysis, we used Enrichr (https://amp.pharm.mssm.edu/Enrichr/), a useful online tool for gene annotation. Terms retrieved with a p-value < 0.05 were considered to be statistically significant.

Next, to investigate the common functions of candidate m6A regulators in EU and EC samples, the DEGs identified between the low and high expression groups (with a |log2FC| > 0.5 and a p < 0.05) in the EU samples were intersected with those from the EC samples. These intersecting DEGs were further evaluated by Reactome pathway analysis; this analysis was carried out on the Reactome website (https://reactome.org).

### The association between diagnostic m6A regulators and infiltrating immune cells in EMs

To investigate the correlation between diagnostic m6A regulators and infiltrating immune cells in EMs, we performed single-sample gene set enrichment analysis (ssGSEA); this is a method used to evaluate gene set enrichment in a single sample and was carried out using the ‘GSVA’ R package [57]. We used a variety of markers for each type of immune cell in EMs, as described by Vallvé et al. [38]: macrophages type 1 (Mφ1), macrophages type 2 (Mφ2), immature dendritic cells (iDCs), mature dendritic cells (mDCs), Natural killer cells (NK), mast cells (MC), Eosinophils (EN), Neutrophils (NT), B cells, CD8+T cells, CD4+Th17 cells, CD4+Th1 cells, CD4+Th2 cells, Treg cells, NKT cells, and γδ T cells. We made these markers into a ‘.gmt’ file as the reference gene set. The ssGSEA enrichment scores obtained for each type of immune cell were then scaled and compared between the low expression and high expression groups of m6A regulators. Furthermore, we also used the Microenvironment Cell Populations-counter (MCP-counter) method [58] to evaluate the infiltration of immune cells in the low expression and high expression groups of m6A regulators for both EU and EC samples. We also analyzed the relationship between m6A regulators and immune infiltration cells by Spearman correlation; p < 0.05 was considered to be statistically significant.

### Potential regulatory mechanisms of diagnostic m6A regulators in EMs

Transcription factors (TFs) play a critical role in the regulation of gene expression. The dysregulation of TFs was frequently described in EMs [1]. To explore the potential regulatory mechanisms of diagnostic m6A regulators in EMs, we predicted TFs and constructed a gene-TF network using Network Analyst version 3.0 (https://www.networkanalyst.ca/) using the JASPAR database (http://jaspar.genereg.net/) as a source. The Gene-TF network was then visualized by software Cytoscape [59]. The expression levels of TFs and their correlation with diagnostic m6A regulators were then examined in both training (‘Kruskal.test’) and validation datasets (‘DEseq2’ package); adjusted p-value < 0.05 was considered to be statistically significant.

## Supplementary Material

Supplementary Figures

Supplementary Table 1

Supplementary Tables 2, 3 and 4
