# Supplementary material for: Exploring diagnostic m6A regulators in endometriosis
Source: Aging (Albany NY). 2020 Nov 24;12(24):25916–38. doi: 10.18632/aging.202163 (PMC7803542; doi:10.18632/aging.202163)
Supplement: Supplementary Figures [file aging-12-202163-s001.pdf]

SUPPLEMENTARY FIGURES

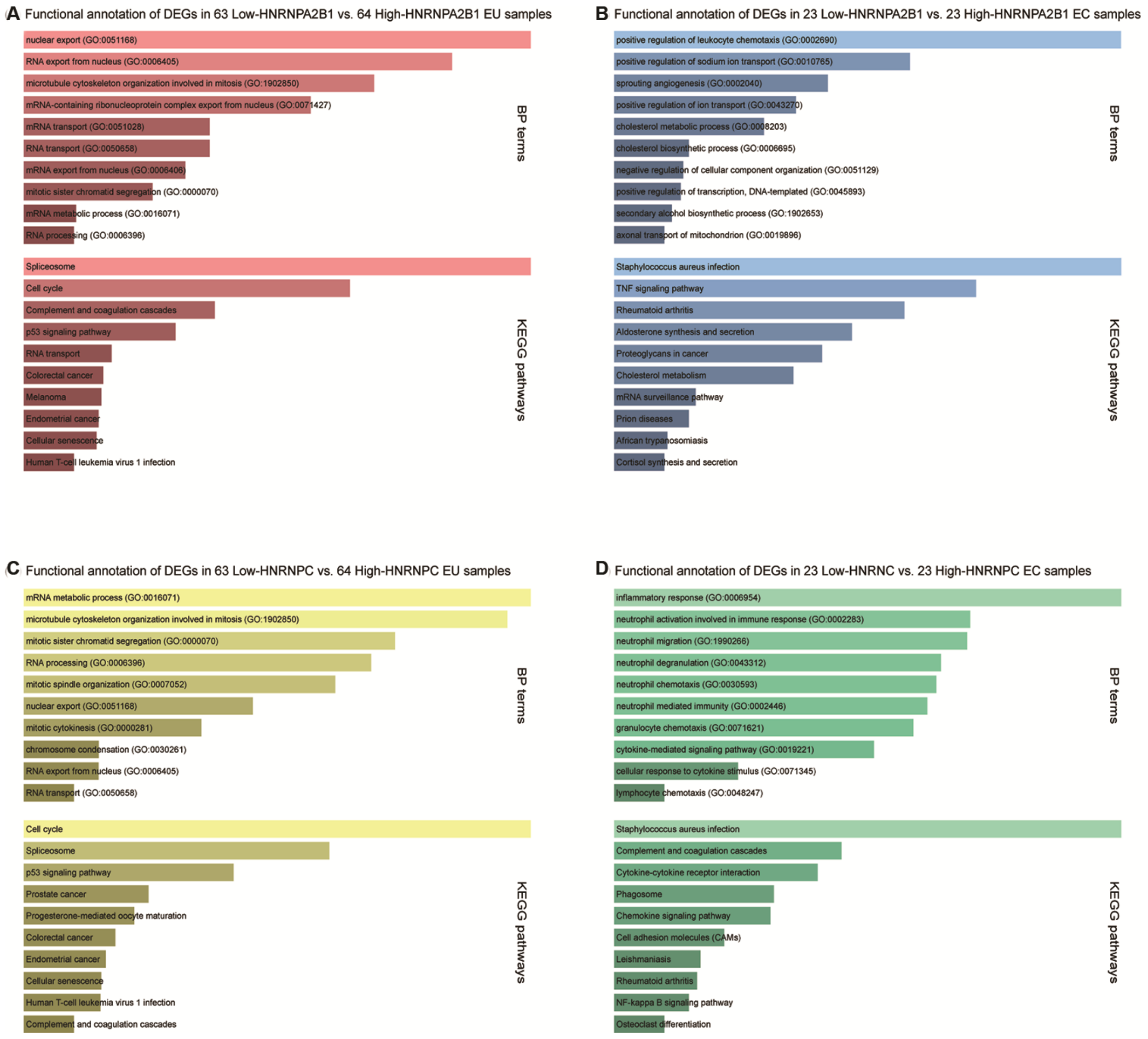

Supplementary Figure 1. The GO and KEGG analysis of DEGs in low-expression vs. high-expression of HNRNPA2B1 or HNRNPC EU and EC samples. The top 10 enriched BP terms and KEGG pathways of DEGs in low-HNRNPA2B1 vs. high-HNRNPA2B1 EU (**A**) and EC (**B**) samples. The top 10 enriched BP terms and KEGG pathways of DEGs in low-HNRNPC vs. high- HNRNPC EU (**C**) and EC (**D**) samples. The length of the horizontal bar represents the Enrihcr combined score of enriched entries. All entries were ranked by p-value in the ascending order. GO, gene ontology; KEGG, Kyoto Encyclopedia of Genes and Genomes; DEG, differentially expressed genes; EU, e utopic endometrium; EC, ectopic endometrium.

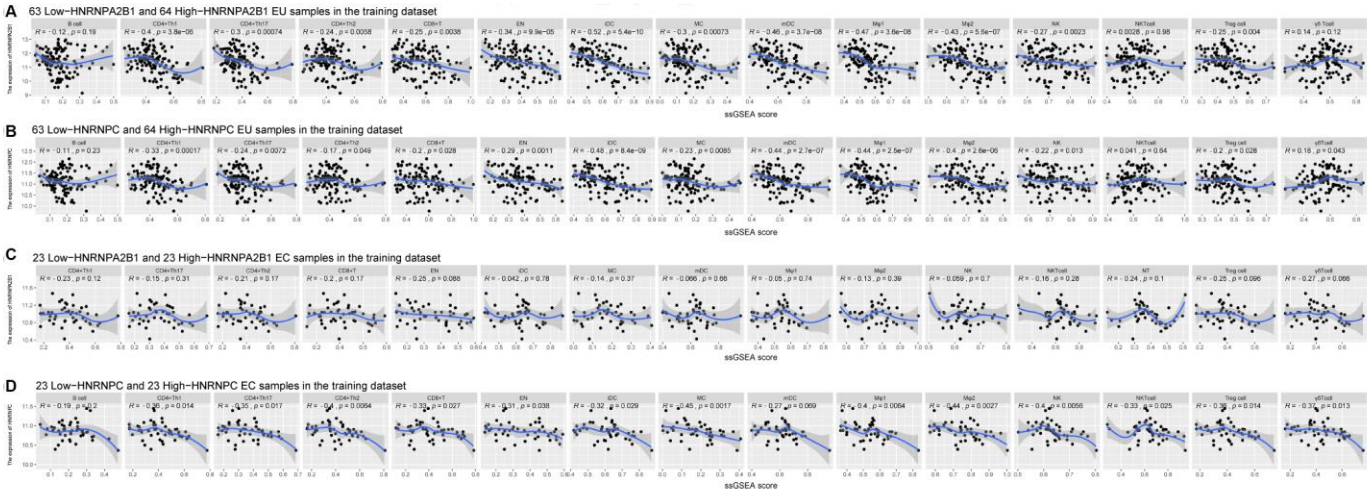

Supplementary Figure 2. The correlation analysis of HNRNPA2B1 and HNRNPC with infiltrating immune cells in EMs. The Spearman correlation analysis of the expression of HNRNPA2B1 and ssGSEA score of 15 other kinds of immune cells in the EU (**A**) and EC (**C**) samples. The Spearman correlation analysis of the expression of HNRNPC and ssGSEA score of 15 other kinds of immune cells in the EU (**B**) and EC (**D**) samples. Mφ1, macrophages type 1; Mφ2, macrophages type 2; iDC, immature dendritic cells; mDC, mature dendritic cells; NK, Natural killer cells; MC, mast cells; EN, Eosinophils; NT, Neutrophils. * $p < 0.05$; ** $p < 0.01$; *** $p < 0.001$.

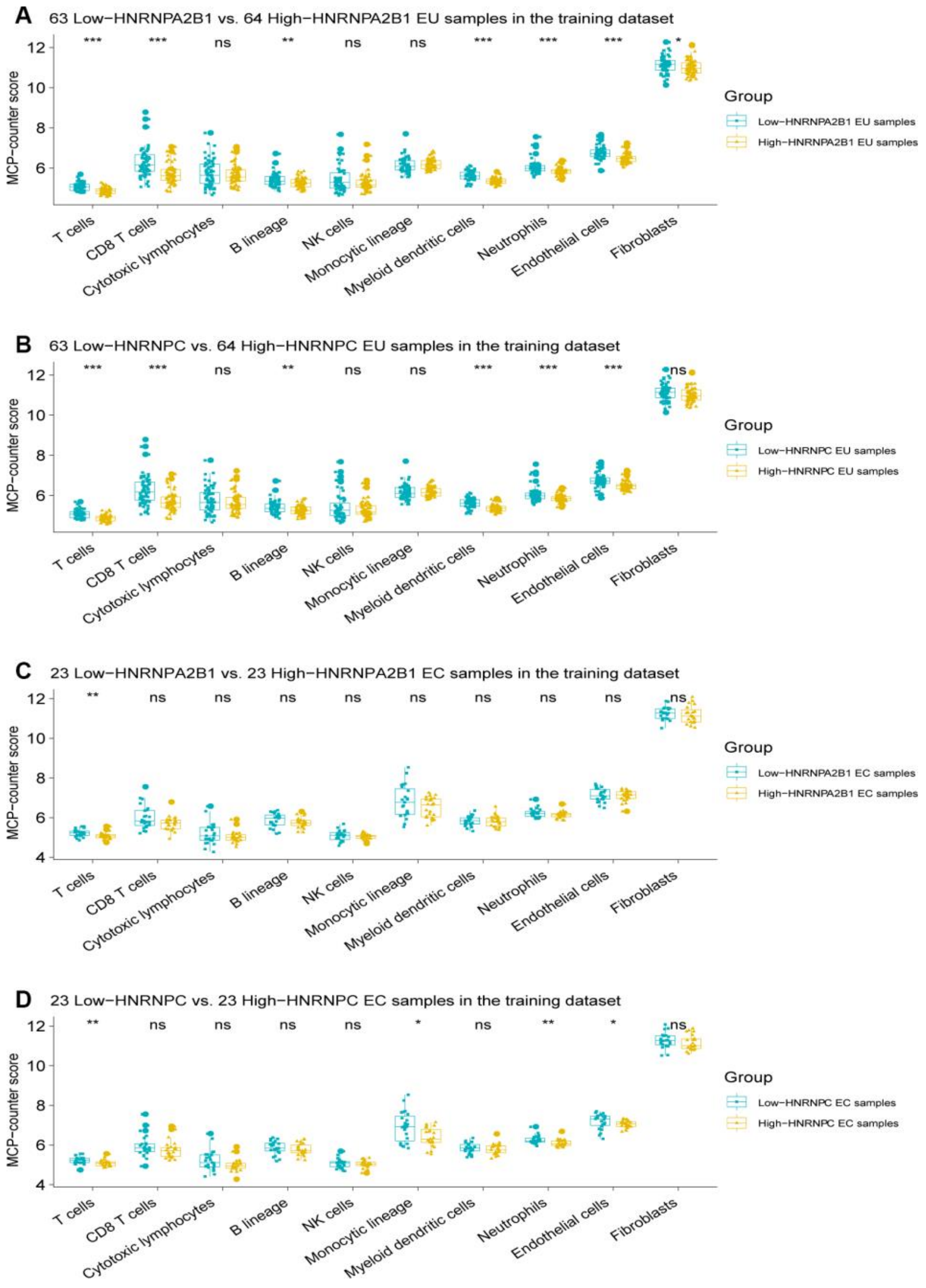

Supplementary Figure 3. The association of HNRNPA2B1 and HNRNPC with infiltrating immune cells in EMs. (**A**–**D**) Differentially expressed MCP-counter scores of several kinds of immune cells between low-HNRNPA2B1 vs. high-HNRNPA2B1 EU samples, and low-HNRNPC vs. high-HNRNPC EU samples, low-HNRNPA2B1 vs. high-HNRNPA2B1 EC samples, and low-HNRNPC vs. high-HNRNPC EC samples ('Wilcox. Test'). EMs, endometriosis; MCP, Microenvironment Cell Populations; EU, eutopic endometrium; EC, ectopic endometrium. NS - not significant, * $p < 0.05$; ** $p < 0.01$, *** $p < 0.001$.

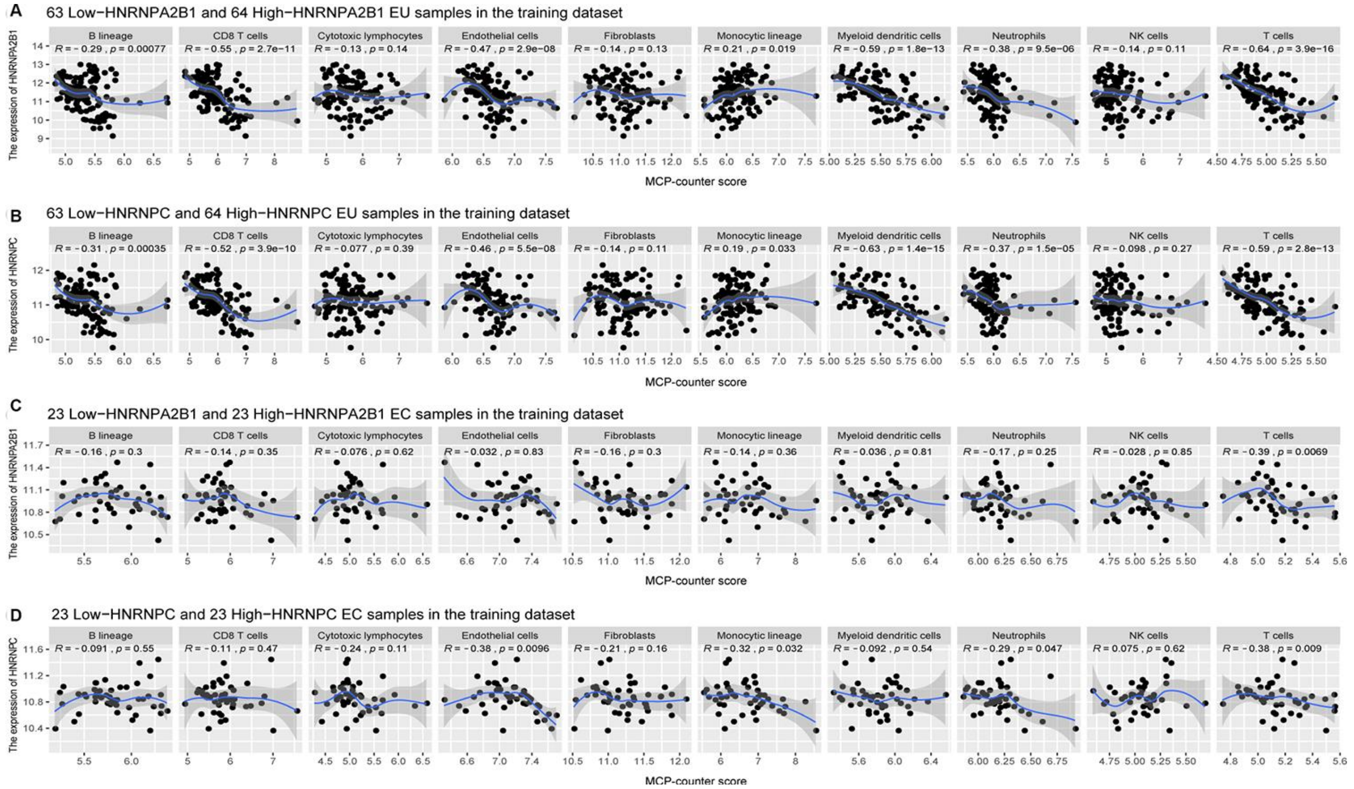

Supplementary Figure 4. The correlation analysis of HNRNPA2B1 and HNRNPC with infiltrating immune cells in EMs. The Spearman correlation analysis of the expression of HNRNPA2B1 and the MCP-counter scores of several kinds of immune cells in the EU (**A**) and EC (**C**) samples. The Spearman correlation analysis of the expression of HNRNPC and the MCP-counter scores of several kinds of immune cells in the EU (**B**) and EC (**D**) samples. MCP, Microenvironment Cell Populations; EU, eutopic endometrium; EC, ectopic endometrium.
