# Supplementary material for: Exploring diagnostic m6A regulators in endometriosis
Source: Aging (Albany NY). 2020 Nov 24;12(24):25916–38. doi: 10.18632/aging.202163 (PMC7803542; doi:10.18632/aging.202163)
Supplement: Supplementary Table 1 [file aging-12-202163-s002.docx]

| **Supplementary Table 1. Clinical information of the retrieved EMs microarray datasets** | | | | | | | | |
| --- | --- | --- | --- | --- | --- | --- | --- | --- |
| **NO.** | **Sample ID** | **Diagnoses** | **Group** | **Batch** | **r-AFS Stage** | **Cycle-phase** | **Race** | **Age(y)** |
| 1 | GSM1256720 | Non-endometriosis | normal | GSE51981 | NA | Proliferative | Caucasian | 39 |
| 2 | GSM1256721 | Non-endometriosis | normal | GSE51981 | NA | Secretory | Black | 33 |
| 3 | GSM1256722 | Non-endometriosis | normal | GSE51981 | NA | Proliferative | Caucasian | 31 |
| 4 | GSM1256723 | Non-endometriosis | normal | GSE51981 | NA | Proliferative | Caucasian | 29 |
| 5 | GSM1256724 | Non-endometriosis | normal | GSE51981 | NA | Secretory | Black | 31 |
| 6 | GSM1256725 | Non-endometriosis | normal | GSE51981 | NA | Proliferative | Caucasian | 29 |
| 7 | GSM1256726 | Non-endometriosis | normal | GSE51981 | NA | Secretory | Caucasian | 32 |
| 8 | GSM1256727 | Non-endometriosis | normal | GSE51981 | NA | Secretory | Caucasian | 33 |
| 9 | GSM1256728 | Non-endometriosis | normal | GSE51981 | NA | Secretory | Black | 35 |
| 10 | GSM1256729 | Non-endometriosis | normal | GSE51981 | NA | Proliferative | Caucasian | 38 |
| 11 | GSM1256730 | Non-endometriosis | normal | GSE51981 | NA | Secretory | Caucasian | 37 |
| 12 | GSM1256731 | Non-endometriosis | normal | GSE51981 | NA | Proliferative | Caucasian | 25 |
| 13 | GSM1256732 | Non-endometriosis | normal | GSE51981 | NA | Secretory | Caucasian | 35 |
| 14 | GSM1256733 | Non-endometriosis | normal | GSE51981 | NA | Proliferative | Caucasian | 31 |
| 15 | GSM1256734 | Non-endometriosis | normal | GSE51981 | NA | Proliferative | Caucasian | 37 |
| 16 | GSM1256766 | Non-endometriosis | normal | GSE51981 | NA | Secretory | Caucasian | 30 |
| 17 | GSM1256767 | Non-endometriosis | normal | GSE51981 | NA | Secretory | Caucasian | 34 |
| 18 | GSM1256768 | Non-endometriosis | normal | GSE51981 | NA | Secretory | Black | 23 |
| 19 | GSM1256769 | Non-endometriosis | normal | GSE51981 | NA | Secretory | Caucasian | 33 |
| 20 | GSM1256770 | Non-endometriosis | normal | GSE51981 | NA | Proliferative | Caucasian | 31 |
| 21 | GSM1256771 | Non-endometriosis | normal | GSE51981 | NA | Proliferative | Caucasian | 32 |
| 22 | GSM1256772 | Non-endometriosis | normal | GSE51981 | NA | Proliferative | Caucasian | 34 |
| 23 | GSM1256784 | Non-endometriosis | normal | GSE51981 | NA | Proliferative | Caucasian | 23 |
| 24 | GSM1256785 | Non-endometriosis | normal | GSE51981 | NA | Proliferative | Black | 28 |
| 25 | GSM1256786 | Non-endometriosis | normal | GSE51981 | NA | Proliferative | Caucasian | 29 |
| 26 | GSM1256787 | Non-endometriosis | normal | GSE51981 | NA | Secretory | NA | 28 |
| 27 | GSM1256788 | Non-endometriosis | normal | GSE51981 | NA | Proliferative | Asian | 23 |
| 28 | GSM1256791 | Non-endometriosis | normal | GSE51981 | NA | Secretory | Caucasian | 36 |
| 29 | GSM1256793 | Non-endometriosis | normal | GSE51981 | NA | Proliferative | Caucasian | 31 |
| 30 | GSM1256796 | Non-endometriosis | normal | GSE51981 | NA | Proliferative | Asian | 37 |
| 31 | GSM1256797 | Non-endometriosis | normal | GSE51981 | NA | Secretory | Caucasian | 37 |
| 32 | GSM1256798 | Non-endometriosis | normal | GSE51981 | NA | Proliferative | Hispanic | 40 |
| 33 | GSM1256799 | Non-endometriosis | normal | GSE51981 | NA | Proliferative | Asian | 36 |
| 34 | GSM1256800 | Non-endometriosis | normal | GSE51981 | NA | Proliferative | Asian | 39 |
| 35 | Pat_01_Endometrium | peritoneal endometriosis (PE) | eutopic | MTAB694 | mod/severe | Proliferative | NA | 40 |
| 36 | Pat_02_Endometrium | peritoneal endometriosis (PE) | eutopic | MTAB694 | min/mild | Secretory | NA | 43 |
| 37 | Pat_03_Endometrium | peritoneal endometriosis (PE) | eutopic | MTAB694 | min/mild | Proliferative | NA | 33 |
| 38 | Pat_04_Endometrium | peritoneal endometriosis (PE) | eutopic | MTAB694 | min/mild | Secretory | NA | 23 |
| 39 | Pat_05_Endometrium | peritoneal endometriosis (PE) | eutopic | MTAB694 | mod/severe | Proliferative | NA | 29 |
| 40 | Pat_06_Endometrium | peritoneal endometriosis (PE) | eutopic | MTAB694 | min/mild | Secretory | NA | 25 |
| 41 | Pat_08_Endometrium | peritoneal endometriosis (PE) | eutopic | MTAB694 | min/mild | Secretory | NA | 52 |
| 42 | Pat_09_Endometrium | peritoneal endometriosis (PE) | eutopic | MTAB694 | mod/severe | Proliferative | NA | 30 |
| 43 | Pat_10_Endometrium | peritoneal endometriosis (PE) | eutopic | MTAB694 | min/mild | Proliferative | NA | 30 |
| 44 | Pat_11_Endometrium | peritoneal endometriosis (PE) | eutopic | MTAB694 | min/mild | Secretory | NA | 37 |
| 45 | Pat_12_Endometrium | peritoneal endometriosis (PE) | eutopic | MTAB694 | min/mild | Proliferative | NA | 35 |
| 46 | Pat_13_Endometrium | peritoneal endometriosis (PE) | eutopic | MTAB694 | min/mild | Secretory | NA | 35 |
| 47 | Pat_14_Endometrium | peritoneal endometriosis (PE) | eutopic | MTAB694 | min/mild | Secretory | NA | 38 |
| 48 | Pat_15_Endometrium | peritoneal endometriosis (PE) | eutopic | MTAB694 | min/mild | Proliferative | NA | 29 |
| 49 | Pat_16_Endometrium | peritoneal endometriosis (PE) | eutopic | MTAB694 | min/mild | Secretory | NA | 33 |
| 50 | Pat_18_Endometrium | peritoneal endometriosis (PE) | eutopic | MTAB694 | min/mild | Proliferative | NA | 47 |
| 51 | Pat_25_Endometrium | peritoneal endometriosis (PE) | eutopic | MTAB694 | mod/severe | Secretory | NA | 36 |
| 52 | GSM175776 | ovarian endometriosis (OE) | eutopic | GSE7305 | NA | Proliferative | Caucasian | NA |
| 53 | GSM175777 | ovarian endometriosis (OE) | eutopic | GSE7305 | NA | Proliferative | Caucasian | NA |
| 54 | GSM175778 | ovarian endometriosis (OE) | eutopic | GSE7305 | NA | Secretory | Caucasian | NA |
| 55 | GSM175779 | ovarian endometriosis (OE) | eutopic | GSE7305 | NA | Secretory | Caucasian | NA |
| 56 | GSM175780 | ovarian endometriosis (OE) | eutopic | GSE7305 | NA | Proliferative | Caucasian | NA |
| 57 | GSM175781 | ovarian endometriosis (OE) | eutopic | GSE7305 | NA | Proliferative | Caucasian | NA |
| 58 | GSM175782 | ovarian endometriosis (OE) | eutopic | GSE7305 | NA | Proliferative | Caucasian | NA |
| 59 | GSM175783 | ovarian endometriosis (OE) | eutopic | GSE7305 | NA | Proliferative | Caucasian | NA |
| 60 | GSM175784 | ovarian endometriosis (OE) | eutopic | GSE7305 | NA | Proliferative | Caucasian | NA |
| 61 | GSM175785 | ovarian endometriosis (OE) | eutopic | GSE7305 | NA | Proliferative | Caucasian | NA |
| 62 | GSM1256653 | Extensive peritoneal endometriosis, pelvic adhesions, chronic pelvic pain | eutopic | GSE51981 | min/mild | Secretory | Asian | 36 |
| 63 | GSM1256654 | Infertility; endometrioma, hydrosalpinx, | eutopic | GSE51981 | min/mild | Secretory | Caucasian | 41 |
| 64 | GSM1256655 | Peritoneal endometriosis, chronic pelvic pain | eutopic | GSE51981 | min/mild | Proliferative | Caucasian | 33 |
| 65 | GSM1256656 | Peritoneal endometriosis chronic pelvic pain | eutopic | GSE51981 | min/mild | Secretory | Caucasian | 42 |
| 66 | GSM1256657 | Peritoneal endometriosis, chronic pelvic pain, fibroids, infertility | eutopic | GSE51981 | min/mild | Secretory | NA | 32 |
| 67 | GSM1256658 | Peritoneal endometriosis, infertility | eutopic | GSE51981 | min/mild | Secretory | Caucasian | 39 |
| 68 | GSM1256659 | Peritoneal endometriosis, bilateral endometriomas, chronic pelvic pain | eutopic | GSE51981 | min/mild | Secretory | Caucasian | 27 |
| 69 | GSM1256660 | Peritoneal endometriosis, bilateral endometriomas, chronic pelvic pain | eutopic | GSE51981 | min/mild | Proliferative | Caucasian | 37 |
| 70 | GSM1256661 | Extensive peritoneal endometriosis, extensive adhesions, chronic pelvic pain | eutopic | GSE51981 | min/mild | Secretory | Black | 41 |
| 71 | GSM1256662 | Extensive peritoneal endometriosis, extensive adhesions, chronic pelvic pain | eutopic | GSE51981 | min/mild | Secretory | Asian Indian | 34 |
| 72 | GSM1256663 | Extensive peritoneal endometriosis, adhesions, hydrosalpinx, chronic pelvic pain | eutopic | GSE51981 | min/mild | Proliferative | Caucasian | 46 |
| 73 | GSM1256664 | Extensive endometriosis, adhesions, endometrioma, hydro, chronic pelvic pain, fibroid | eutopic | GSE51981 | min/mild | Proliferative | Caucasian | 27 |
| 74 | GSM1256665 | Peritoneal endometriosis, dysmenorrhea, fibroid, chronic pelvic pain | eutopic | GSE51981 | min/mild | Secretory | Caucasian | 45 |
| 75 | GSM1256666 | Peritoneal endometriosis, endometrioma, adhesions, chronic pelvic pain | eutopic | GSE51981 | min/mild | Proliferative | Caucasian | 43 |
| 76 | GSM1256667 | RectovagiNon-endometriosisl, peritoneal endometriosis, endometrioma, chronic pelvic pain | eutopic | GSE51981 | min/mild | Secretory | Caucasian | 38 |
| 77 | GSM1256668 | RectovagiNon-endometriosisl, peritoneal endometriosis, chronic pelvic pain | eutopic | GSE51981 | min/mild | Secretory | Caucasian | 37 |
| 78 | GSM1256669 | Peritoneal endometriosis, endometrioma, infertility | eutopic | GSE51981 | min/mild | Secretory | Caucasian | 33 |
| 79 | GSM1256670 | Peritoneal endometriosis, chronic pelvic pain, adhesions | eutopic | GSE51981 | min/mild | Secretory | Caucasian | 38 |
| 80 | GSM1256671 | Peritoneal endometriosis, endometrioma, chronic pelvic pain | eutopic | GSE51981 | min/mild | Secretory | Caucasian | 48 |
| 81 | GSM1256672 | Peritoneal endometriosis, infertility | eutopic | GSE51981 | min/mild | Secretory | Caucasian | 36 |
| 82 | GSM1256673 | Extensive endometriosis, adhesions, endometrioma, chronic pelvic pain, adenomyosis | eutopic | GSE51981 | min/mild | Proliferative | Caucasian | 28 |
| 83 | GSM1256674 | Peritoneal endometriosis, chronic pelvic pain, spotting | eutopic | GSE51981 | min/mild | Secretory | NA | 25 |
| 84 | GSM1256675 | Peritoneal endometriosis, chronic pelvic pain, spotting | eutopic | GSE51981 | min/mild | Secretory | Asian | 34 |
| 85 | GSM1256676 | Extensive peritoneal endometriosis, chronic pelvic pain | eutopic | GSE51981 | min/mild | Secretory | Caucasian | 28 |
| 86 | GSM1256677 | Peritoneal endometriosis, cervical stenosis, fibroid, appendiceal adhesions | eutopic | GSE51981 | min/mild | Secretory | NA | 31 |
| 87 | GSM1256678 | Endometrioma/peritoneal endometriosis, extensive adhesions, chronic pelvic pain | eutopic | GSE51981 | min/mild | Proliferative | NA | 42 |
| 88 | GSM1256679 | Extensive peritoneal, rectovagiNon-endometriosisl endometriosis, adhesions, chronic pelvic pain | eutopic | GSE51981 | min/mild | Proliferative | Caucasian | 32 |
| 89 | GSM1256680 | Extensive endometriosis, adhesions, fibroids | eutopic | GSE51981 | min/mild | Proliferative | Asian | 50 |
| 90 | GSM1256681 | Extensive peritoneal rectovagiNon-endometriosisl endometriosis, adhesions, chronic pelvic pain | eutopic | GSE51981 | mod/severe | Secretory | Asian | 41 |
| 91 | GSM1256682 | Peritoneal endometriosis, infertiity, chronic pelvic pain | eutopic | GSE51981 | mod/severe | Proliferative | NA | 50 |
| 92 | GSM1256683 | Extensive pelvic adhesions/endometriosis | eutopic | GSE51981 | mod/severe | Proliferative | Caucasian | 31 |
| 93 | GSM1256684 | Peritoneal endometriosis, fibroid, dysmenorrhea, chronic pelvic pain | eutopic | GSE51981 | mod/severe | Proliferative | Caucasian | 25 |
| 94 | GSM1256685 | Peritoneal endometriosis, fibroid, infertility | eutopic | GSE51981 | mod/severe | Proliferative | Caucasian | 38 |
| 95 | GSM1256686 | Peritoneal endometriosis, dysmenorrhea, chronic pelvic pain | eutopic | GSE51981 | mod/severe | Proliferative | Caucasian | 26 |
| 96 | GSM1256687 | Extensive peritoneal endometriosis, adhesions, symptomatic fibroids | eutopic | GSE51981 | mod/severe | Secretory | Caucasian | 36 |
| 97 | GSM1256688 | Peritoneal endometriosis, hydrosalpinx | eutopic | GSE51981 | mod/severe | Secretory | Caucasian | 48 |
| 98 | GSM1256689 | Peritoneal endometriosis, chronic pelvic pain | eutopic | GSE51981 | mod/severe | Proliferative | NA | 39 |
| 99 | GSM1256690 | Peritoneal endometriosis, chronic pelvic pain | eutopic | GSE51981 | mod/severe | Secretory | Caucasian | 37 |
| 100 | GSM1256691 | Peritoneal endometriosis, adhesions, fibroid, chronic pelvic pain | eutopic | GSE51981 | mod/severe | Proliferative | NA | 42 |
| 101 | GSM1256692 | Peritoneal endometriosis, adhesions, fibroid, chronic pelvic pain | eutopic | GSE51981 | mod/severe | Secretory | Black | 29 |
| 102 | GSM1256693 | Peritoneal endometriosis, pelvic prolapse | eutopic | GSE51981 | mod/severe | Proliferative | Caucasian | 42 |
| 103 | GSM1256694 | Peritoneal endometriosis, chronic pelvic pain, dysfunctioNon-endometriosisl uterine bleeding | eutopic | GSE51981 | mod/severe | Secretory | Caucasian | 30 |
| 104 | GSM1256695 | extensive pelvic/vagiNon-endometriosisl endometriosis/adhesions, chronic pelvic pain | eutopic | GSE51981 | mod/severe | Secretory | Caucasian | 37 |
| 105 | GSM1256696 | Peritoneal endometriosis chronic pelvic pain, adhesions | eutopic | GSE51981 | mod/severe | Secretory | NA | 25 |
| 106 | GSM1256697 | Extensive endometriosis and adhesions, bilateral endometriomas, chronic pelvic pain | eutopic | GSE51981 | mod/severe | Secretory | Caucasian | 40 |
| 107 | GSM1256698 | Peritoneal endometriosis, dysmenorrhea, unexplained infertility | eutopic | GSE51981 | mod/severe | Proliferative | Caucasian | 29 |
| 108 | GSM1256699 | Extensive endometriosis, adhesions, endometrioma | eutopic | GSE51981 | mod/severe | Proliferative | Caucasian | 37 |
| 109 | GSM1256700 | Peritoneal endometriosis, chronic pelvic pain | eutopic | GSE51981 | mod/severe | Secretory | Caucasian | 35 |
| 110 | GSM1256701 | Extensive peritoneal endometriosis, adhesions, chronic pelvic pain, endometrioma | eutopic | GSE51981 | mod/severe | Secretory | Asian | 24 |
| 111 | GSM1256702 | Peritoneal endometriosis, multiple fibroids, chronic pelvic pain | eutopic | GSE51981 | mod/severe | Proliferative | Caucasian | 35 |
| 112 | GSM1256703 | Extensive peritoneal endometriosis/adhesions, chronic pelvic pain | eutopic | GSE51981 | mod/severe | Proliferative | Hispanic | 22 |
| 113 | GSM1256704 | Extensive endometriosis, adhesions, chronic pelvic pain, obliterated cul de sac | eutopic | GSE51981 | mod/severe | Secretory | Caucasian | 39 |
| 114 | GSM1256705 | Peritoneal endometriosis, chronic pelvic pain, uterine fibroids | eutopic | GSE51981 | mod/severe | Secretory | NA | 36 |
| 115 | GSM1256706 | Peritoneal endometriosis, infertility | eutopic | GSE51981 | mod/severe | Proliferative | Caucasian | 45 |
| 116 | GSM1256707 | Extensive peritoneal endometriosis, chronic pelvic pain, fibroid | eutopic | GSE51981 | mod/severe | Proliferative | Caucasian | 34 |
| 117 | GSM1256708 | Peritoneal endometriosis, chronic pelvic pain, fibroids, corpus luteum cyst | eutopic | GSE51981 | mod/severe | Secretory | Caucasian | 38 |
| 118 | GSM1256709 | Extensive pelvic endometriosis and adhesions, chronic pelvic pain | eutopic | GSE51981 | mod/severe | Proliferative | Caucasian | 43 |
| 119 | GSM1256710 | Extensive peritoneal endometriosis, extensive pelvic adhesions, chronic deep pelvic pain | eutopic | GSE51981 | mod/severe | Secretory | Caucasian | 35 |
| 120 | GSM1256711 | Extensive peritoneal endometriosis, pelvic adhesions, chronic pelvic pain, infertility | eutopic | GSE51981 | mod/severe | Secretory | Caucasian | 32 |
| 121 | GSM1256712 | Extensive peritoneal endometriosis, adhesions, hydrosalpinges, chronic pelvic pain | eutopic | GSE51981 | mod/severe | Secretory | Caucasian | 20 |
| 122 | GSM1256713 | Peritoneal endometriosis, chronic pelvic pain, endometrial polyps | eutopic | GSE51981 | mod/severe | Secretory | Caucasian | 35 |
| 123 | GSM1256714 | Extensive endometriosis, adhesions, endometrioma, fibroids, chronic pelvic pain | eutopic | GSE51981 | mod/severe | Secretory | Caucasian | 37 |
| 124 | GSM1256715 | Peritoneal endometriosis, chronic pelvic pain | eutopic | GSE51981 | mod/severe | Secretory | Caucasian | 38 |
| 125 | GSM1256716 | Extensive endometriosis, adhesions, fibroids, endometriomas | eutopic | GSE51981 | mod/severe | Secretory | Caucasian | 39 |
| 126 | GSM1256717 | Extensive endometriosis, adhesions, chronic pelvic pain, infertility | eutopic | GSE51981 | mod/severe | Secretory | Caucasian | 44 |
| 127 | GSM1256718 | Peritoneal endometriosis, adhesions, chronic pelvic pain | eutopic | GSE51981 | mod/severe | Proliferative | Caucasian | 27 |
| 128 | GSM1256719 | Extensive peritoneal endometriosis, endometrioma | eutopic | GSE51981 | mod/severe | Proliferative | Caucasian | 35 |
| 129 | GSM1256773 | Endometriosis, left endometrioma, symptomatic uterine fibroids, chronic cervicitis | eutopic | GSE51981 | mod/severe | Indeterminate | Asian | 31 |
| 130 | GSM1256774 | Peritoneal endometriosis, chronic pelvic pain | eutopic | GSE51981 | mod/severe | Secretory | Caucasian | 26 |
| 131 | GSM1256775 | Endometriosis of fallopian tube, symptomatic fibroids, endocervical polyp | eutopic | GSE51981 | mod/severe | Indeterminate | Caucasian | 46 |
| 132 | GSM1256776 | Peritoneal endometriosis, chronic pelvic pain | eutopic | GSE51981 | mod/severe | Secretory | Caucasian | 44 |
| 133 | GSM1256777 | Extensive peritoneal endometriosis, fibroids, dyspareunia, chronic pelvic pain | eutopic | GSE51981 | mod/severe | Proliferative | Caucasian | 32 |
| 134 | GSM1256778 | Peritoneal endometriosis, fibroids, chronic pelvic pain | eutopic | GSE51981 | mod/severe | Secretory | Asian | 31 |
| 135 | GSM1256779 | Endometrioma, peritoneal endometriosis, endometrial polyp | eutopic | GSE51981 | mod/severe | Proliferative | Asian | 41 |
| 136 | GSM1256780 | Extensive peritoneal endometriosis, adhesions, adnexal mass, chronic pelvic pain | eutopic | GSE51981 | mod/severe | Secretory | NA | 29 |
| 137 | GSM1256781 | Extensive peritoneal endometriosis, adhesions, chronic pelvic pain, infertility | eutopic | GSE51981 | mod/severe | Secretory | NA | 27 |
| 138 | GSM1256782 | Extensive peritoneal endometriosis, adhesions, chronic pelvic pain, infertility | eutopic | GSE51981 | mod/severe | Proliferative | NA | 39 |
| 139 | GSM176039 | ovarian endometriosis (OE) | eutopic | GSE7307 | NA | NA | NA | NA |
| 140 | GSM176137 | ovarian endometriosis (OE) | eutopic | GSE7307 | NA | NA | NA | NA |
| 141 | GSM176100 | ovarian endometriosis (OE) | eutopic | GSE7307 | NA | NA | NA | NA |
| 142 | GSM176101 | ovarian endometriosis (OE) | eutopic | GSE7307 | NA | NA | NA | NA |
| 143 | GSM176141 | ovarian endometriosis (OE) | eutopic | GSE7307 | NA | NA | NA | NA |
| 144 | GSM176142 | ovarian endometriosis (OE) | eutopic | GSE7307 | NA | NA | NA | NA |
| 145 | GSM176143 | ovarian endometriosis (OE) | eutopic | GSE7307 | NA | NA | NA | NA |
| 146 | GSM176144 | ovarian endometriosis (OE) | eutopic | GSE7307 | NA | NA | NA | NA |
| 147 | GSM176145 | ovarian endometriosis (OE) | eutopic | GSE7307 | NA | NA | NA | NA |
| 148 | GSM176146 | ovarian endometriosis (OE) | eutopic | GSE7307 | NA | NA | NA | NA |
| 149 | GSM176040 | ovarian endometriosis (OE) | eutopic | GSE7307 | NA | NA | NA | NA |
| 150 | GSM176041 | ovarian endometriosis (OE) | eutopic | GSE7307 | NA | NA | NA | NA |
| 151 | GSM176099 | ovarian endometriosis (OE) | eutopic | GSE7307 | NA | NA | NA | NA |
| 152 | GSM176043 | ovarian endometriosis (OE) | eutopic | GSE7307 | NA | NA | NA | NA |
| 153 | GSM176097 | ovarian endometriosis (OE) | eutopic | GSE7307 | NA | NA | NA | NA |
| 154 | GSM176098 | ovarian endometriosis (OE) | eutopic | GSE7307 | NA | NA | NA | NA |
| 155 | GSM176319 | ovarian endometriosis (OE) | eutopic | GSE7307 | NA | NA | NA | NA |
| 156 | GSM176093 | ovarian endometriosis (OE) | eutopic | GSE7307 | NA | NA | NA | NA |
| 157 | GSM176094 | ovarian endometriosis (OE) | eutopic | GSE7307 | NA | NA | NA | NA |
| 158 | GSM176095 | ovarian endometriosis (OE) | eutopic | GSE7307 | NA | NA | NA | NA |
| 159 | GSM176096 | ovarian endometriosis (OE) | eutopic | GSE7307 | NA | NA | NA | NA |
| 160 | GSM176127 | ovarian endometriosis (OE) | eutopic | GSE7307 | NA | NA | NA | NA |
| 161 | GSM176132 | ovarian endometriosis (OE) | eutopic | GSE7307 | NA | NA | NA | NA |
| 162 | Pat_01_Lesion | peritoneal endometriosis (PE) | ectopic | MTAB694 | severe | Proliferative | NA | 40 |
| 163 | Pat_02_Lesion | peritoneal endometriosis (PE) | ectopic | MTAB694 | mild | Secretory | NA | 43 |
| 164 | Pat_03_Lesion | peritoneal endometriosis (PE) | ectopic | MTAB694 | mild | Proliferative | NA | 33 |
| 165 | Pat_04_Lesion | peritoneal endometriosis (PE) | ectopic | MTAB694 | mild | Secretory | NA | 23 |
| 166 | Pat_05_Lesion | peritoneal endometriosis (PE) | ectopic | MTAB694 | severe | Proliferative | NA | 29 |
| 167 | Pat_06_Lesion | peritoneal endometriosis (PE) | ectopic | MTAB694 | mild | Secretory | NA | 25 |
| 168 | Pat_07_Lesion | peritoneal endometriosis (PE) | ectopic | MTAB694 | severe | Secretory | NA | 36 |
| 169 | Pat_08_Lesion | peritoneal endometriosis (PE) | ectopic | MTAB694 | mild | Secretory | NA | 52 |
| 170 | Pat_09_Lesion | peritoneal endometriosis (PE) | ectopic | MTAB694 | severe | Proliferative | NA | 30 |
| 171 | Pat_10_Lesion | peritoneal endometriosis (PE) | ectopic | MTAB694 | mild | Proliferative | NA | 30 |
| 172 | Pat_11_Lesion | peritoneal endometriosis (PE) | ectopic | MTAB694 | mild | Secretory | NA | 37 |
| 173 | Pat_12_Lesion | peritoneal endometriosis (PE) | ectopic | MTAB694 | mild | Proliferative | NA | 35 |
| 174 | Pat_13_Lesion | peritoneal endometriosis (PE) | ectopic | MTAB694 | mild | Secretory | NA | 35 |
| 175 | Pat_14_Lesion | peritoneal endometriosis (PE) | ectopic | MTAB694 | mild | Secretory | NA | 38 |
| 176 | Pat_16_Lesion | peritoneal endometriosis (PE) | ectopic | MTAB694 | mild | Secretory | NA | 33 |
| 177 | Pat_17_Lesion | peritoneal endometriosis (PE) | ectopic | MTAB694 | mild | Proliferative | NA | 21 |
| 178 | Pat_18_Lesion | peritoneal endometriosis (PE) | ectopic | MTAB694 | mild | Proliferative | NA | 47 |
| 179 | Pat_25_Lesion | peritoneal endometriosis (PE) | ectopic | MTAB694 | severe | Secretory | NA | 36 |
| 180 | GSM175766 | ovarian endometriosis (OE) | ectopic | GSE7305 | NA | Proliferative | Caucasian | NA |
| 181 | GSM175767 | ovarian endometriosis (OE) | ectopic | GSE7305 | NA | Proliferative | Caucasian | NA |
| 182 | GSM175768 | ovarian endometriosis (OE) | ectopic | GSE7305 | NA | Secretory | Caucasian | NA |
| 183 | GSM175769 | ovarian endometriosis (OE) | ectopic | GSE7305 | NA | Secretory | Caucasian | NA |
| 184 | GSM175770 | ovarian endometriosis (OE) | ectopic | GSE7305 | NA | Proliferative | Caucasian | NA |
| 185 | GSM175771 | ovarian endometriosis (OE) | ectopic | GSE7305 | NA | Proliferative | Caucasian | NA |
| 186 | GSM175772 | ovarian endometriosis (OE) | ectopic | GSE7305 | NA | Proliferative | Caucasian | NA |
| 187 | GSM175773 | ovarian endometriosis (OE) | ectopic | GSE7305 | NA | Proliferative | Caucasian | NA |
| 188 | GSM175774 | ovarian endometriosis (OE) | ectopic | GSE7305 | NA | Proliferative | Caucasian | NA |
| 189 | GSM175775 | ovarian endometriosis (OE) | ectopic | GSE7305 | NA | Proliferative | Caucasian | NA |
| 190 | GSM175786 | ovarian endometriosis (OE) | ectopic | GSE7307 | NA | NA | NA | NA |
| 191 | GSM176088 | ovarian endometriosis (OE) | ectopic | GSE7307 | NA | NA | NA | NA |
| 192 | GSM176089 | ovarian endometriosis (OE) | ectopic | GSE7307 | NA | NA | NA | NA |
| 193 | GSM176090 | ovarian endometriosis (OE) | ectopic | GSE7307 | NA | NA | NA | NA |
| 194 | GSM176091 | ovarian endometriosis (OE) | ectopic | GSE7307 | NA | NA | NA | NA |
| 195 | GSM176092 | ovarian endometriosis (OE) | ectopic | GSE7307 | NA | NA | NA | NA |
| 196 | GSM176234 | ovarian endometriosis (OE) | ectopic | GSE7307 | NA | NA | NA | NA |
| 197 | GSM176236 | ovarian endometriosis (OE) | ectopic | GSE7307 | NA | NA | NA | NA |
| 198 | GSM176238 | ovarian endometriosis (OE) | ectopic | GSE7307 | NA | NA | NA | NA |
| 199 | GSM176240 | ovarian endometriosis (OE) | ectopic | GSE7307 | NA | NA | NA | NA |
| 200 | GSM175787 | ovarian endometriosis (OE) | ectopic | GSE7307 | NA | NA | NA | NA |
| 201 | GSM175788 | ovarian endometriosis (OE) | ectopic | GSE7307 | NA | NA | NA | NA |
| 202 | GSM176082 | ovarian endometriosis (OE) | ectopic | GSE7307 | NA | NA | NA | NA |
| 203 | GSM176083 | ovarian endometriosis (OE) | ectopic | GSE7307 | NA | NA | NA | NA |
| 204 | GSM176084 | ovarian endometriosis (OE) | ectopic | GSE7307 | NA | NA | NA | NA |
| 205 | GSM176085 | ovarian endometriosis (OE) | ectopic | GSE7307 | NA | NA | NA | NA |
| 206 | GSM176086 | ovarian endometriosis (OE) | ectopic | GSE7307 | NA | NA | NA | NA |
| 207 | GSM176087 | ovarian endometriosis (OE) | ectopic | GSE7307 | NA | NA | NA | NA |
| 208 | GSM150227 | Non-endometriosis | normal | GSE6364 | NA | Secretory | Caucasian | 33 |
| 209 | GSM150226 | Non-endometriosis | normal | GSE6364 | NA | Secretory | Black | 23 |
| 210 | GSM150225 | Non-endometriosis | normal | GSE6364 | NA | Secretory | Caucasian | 34 |
| 211 | GSM150224 | Non-endometriosis | normal | GSE6364 | NA | Secretory | Caucasian | 30 |
| 212 | GSM150219 | ovarian endometriosis, peritoneal endometriosis | eutopic | GSE6364 | NA | Secretory | Caucasian | 26 |
| 213 | GSM150218 | peritoneal endometriosis | eutopic | GSE6364 | NA | Secretory | Caucasian | 31 |
| 214 | GSM150217 | ovarian endometriosis, peritoneal endometriosis | eutopic | GSE6364 | NA | Secretory | NA | 35 |
| 215 | GSM150216 | rectovaginal endometriosis, peritoneal endometriosis | eutopic | GSE6364 | NA | Secretory | Asian | 44 |
| 216 | GSM150215 | rectovaginal endometriosis, peritoneal endometriosis | eutopic | GSE6364 | NA | Secretory | Asian | 39 |
| 217 | GSM150214 | rectovaginal endometriosis, ovarian endometriosis, peritoneal endometriosis | eutopic | GSE6364 | NA | Secretory | Caucasian | 38 |
| 218 | GSM150213 | rectovaginal endometriosis, peritoneal endometriosis | eutopic | GSE6364 | NA | Secretory | Caucasian | 37 |
| 219 | GSM150212 | rectovaginal endometriosis, peritoneal endometriosis | eutopic | GSE6364 | NA | Secretory | Asian | 34 |
| 220 | GSM150211 | rectovaginal endometriosis, peritoneal endometriosis | eutopic | GSE6364 | NA | Secretory | Caucasian | 27 |
| 221 | GSM150207 | rectovaginal endometriosis, ovarian endometriosis, peritoneal endometriosis | eutopic | GSE6364 | NA | Secretory | Black | 35 |
| 222 | GSM150206 | ovarian endometriosis, peritoneal endometriosis | eutopic | GSE6364 | NA | Secretory | NA | 26 |
| 223 | GSM150205 | rectovaginal endometriosis, peritoneal endometriosis | eutopic | GSE6364 | NA | Secretory | Asian | 35 |
| 224 | GSM150204 | ovarian endometriosis, peritoneal endometriosis | eutopic | GSE6364 | NA | Secretory | Caucasian | 37 |
| 225 | GSM150203 | rectovaginal endometriosis, peritoneal endometriosis | eutopic | GSE6364 | NA | Secretory | Asian | 39 |
| 226 | GSM150202 | rectovaginal endometriosis, ovarian endometriosis, peritoneal endometriosis | eutopic | GSE6364 | NA | Secretory | Caucasian | 22 |
| 227 | GSM150201 | Non-endometriosis | normal | GSE6364 | NA | Proliferative | Caucasian | 34 |
| 228 | GSM150199 | Non-endometriosis | normal | GSE6364 | NA | Proliferative | Caucasian | 32 |
| 229 | GSM150198 | Non-endometriosis | normal | GSE6364 | NA | Proliferative | Caucasian | 31 |
| 230 | GSM150195 | ovarian endometriosis, peritoneal endometriosis | eutopic | GSE6364 | NA | Proliferative | Caucasian | 37 |
| 231 | GSM150194 | rectovaginal endometriosis, ovarian endometriosis, peritoneal endometriosis | eutopic | GSE6364 | NA | Proliferative | Caucasian | 39 |
| 232 | GSM150193 | ovarian endometriosis, peritoneal endometriosis | eutopic | GSE6364 | NA | Proliferative | Caucasian | 38 |
| 233 | GSM150192 | peritoneal endometriosis | eutopic | GSE6364 | NA | Proliferative | Caucasian | 37 |
| 234 | GSM150191 | rectovaginal endometriosis, ovarian endometriosis, peritoneal endometriosis | eutopic | GSE6364 | NA | Proliferative | Caucasian | 25 |
| 235 | GSM150190 | ovarian endometriosis, peritoneal endometriosis | eutopic | GSE6364 | NA | Proliferative | Caucasian | 31 |
| **Notes.** EMs, endometriosis; NA, unknown | | |  |  |  |  |  |  |
