# Supplementary material for: Exploring diagnostic m6A regulators in endometriosis
Source: Aging (Albany NY). 2020 Nov 24;12(24):25916–38. doi: 10.18632/aging.202163 (PMC7803542; doi:10.18632/aging.202163)
Supplement: Supplementary Tables 2, 3 and 4 [file aging-12-202163-s003.pdf]

SUPPLEMENTARY TABLES

Supplementary Table 2. The differentially expressed m6A regulators in EMs microarray training datasets.

	EU *vs.* NM (EU/NM)			EC *vs.* EU (EC/EU)		
**Name**	**log2FC**	**P.Value**	**Significance**	**log2FC**	**P.Value**	**Significance**
FTO	-0.2881393	4.45E-07	***	0.10638329	4.86E-02	*
IGF2BP2	-0.2027905	0.13474953	ns	0.26830358	0.02182174	*
YTHDF3	-0.6648875	1.30E-12	***	-0.1571177	3.71E-02	*
METTL3	-0.7270406	3.41E-08	***	-0.2870733	8.12E-03	**
HNRNPA2B1	-1.1033342	2.68E-12	***	-0.4204266	8.87E-04	***
HNRNPC	-0.6459411	5.85E-13	***	-0.2610574	3.83E-04	***
YTHDF1	-0.0809309	0.08952423	ns	-0.150473	0.00046743	***
YTHDF2	-0.717622	7.85E-13	***	-0.5090405	3.51E-10	***
ALKBH5	0.51203452	4.33E-11	***	0.05133396	4.30E-01	ns
IGF2BP1	0.42602254	4.97E-08	***	-0.0651357	2.81E-01	ns
IGF2BP3	0.22887019	2.41E-06	***	0.03520955	4.32E-01	ns
KIAA1429	-0.8709868	5.64E-10	***	0.18583386	1.04E-01	ns
METTL14	-1.1208207	1.49E-15	***	0.15346996	1.74E-01	ns
RBM15	-0.4471974	1.59E-09	***	0.00835992	8.91E-01	ns
RBM15B	0.54463734	5.44E-08	***	-0.0680207	4.23E-01	ns
RBMX	-0.5232512	6.44E-07	***	0.0200392	8.18E-01	ns
WTAP	-0.0742863	0.40828576	ns	0.13260033	0.10140101	ns
YTHDC1	-0.6553325	4.14E-08	***	0.10894221	2.76E-01	ns
YTHDC2	-0.97265	2.10E-09	***	-0.2171349	1.01E-01	ns
ZC3H13	-0.8732528	2.56E-14	***	-0.06219	4.89E-01	ns

Notes. EMs, endometriosis; NM, normal endometrium; EU, eutopic endometrium; EC, ectopic endometrium (lesions).

Supplementary Table 3. Expression of the predicted TFs of HNRNPA2B1 and HNRNPC in EMs microarray training datasets.

Predicted TFs	EU *vs.* NM (EU/NM)			EC *vs.* EU (EC/EU)		
	log2FC	P-value	Adjust P-value	log2FC	P-value	Adjust P-value
SRF	0.937816753	2.93159E-18	3.28601E-16	0.283409283	0.001910008	0.005658289
ELK1	0.90133241	6.81991E-14	1.06718E-12	0.422113788	2.58652E-05	0.000110835
USF2	0.763393179	2.67592E-13	3.36219E-12	0.453637954	2.00651E-08	1.41032E-07
FOXC1	0.682710767	7.52692E-11	4.32427E-10	1.073584182	2.22683E-24	1.30288E-22
HNF4A	0.435592414	7.98433E-10	3.50675E-09	0.181613037	0.005441308	0.014372262
ESR1	-0.808466804	8.64565E-06	1.82956E-05	-2.504983463	5.31072E-33	8.70018E-31
YY1	-0.850791954	1.49028E-16	7.41551E-15	-0.336656416	4.95696E-05	0.000202672
BRCA1	-0.85953267	1.38837E-09	5.75255E-09	-0.923669383	1.73431E-18	4.54871E-17
NFYA	-0.994272907	1.71719E-13	2.31461E-12	-0.57645333	1.86405E-07	1.13978E-06
TP53	1.135162319	5.81717E-13	6.44236E-12	-0.852111512	8.42895E-11	8.35325E-10
E2F1	0.641672849	4.8711E-09	1.79979E-08	-0.269946406	0.002703268	0.007734985
GATA2	0.281679592	0.033456806	0.043654552	-1.855289513	4.34833E-35	9.54705E-33
NRF1	-0.268901017	0.000237194	0.000411798	0.204698814	0.003380947	0.009423378
MEF2A	-0.759867857	2.18514E-07	5.93871E-07	0.357398819	0.004328805	0.011760499
NFIC	0.663084074	1.62464E-06	3.82829E-06	0.141026946	0.211018861	0.322386563
E2F4	0.603168734	5.24982E-09	1.92569E-08	0.004139902	0.95627151	0.972146321
KLF5	0.160903671	0.448097822	0.487765874	-1.03768277	8.15872E-09	6.0839E-08
TFAP2A	0.148974581	0.084063726	0.103761747	0.018125549	0.808173811	0.871486611
SP1	-0.109077553	0.163149652	0.192136592	0.139989122	0.025249554	0.054938676
STAT1	-0.72669564	4.24047E-08	1.30788E-07	-0.019111672	0.861181585	0.909570731
CREB1	-0.733884273	1.31281E-12	1.28017E-11	0.070907852	0.393434483	0.518950464
TEAD1	-1.000429382	7.6307E-11	4.3721E-10	-0.020885116	0.867189737	0.913334644

Notes. EMs, endometriosis; NM, normal endometrium; EU, eutopic endometrium; EC, ectopic endometrium (lesions); TF, transcription factor;

Supplementary Table 4. The validation analysis of dysregulated TFs of HNRNPA2B1 and HNRNPC in GSE105764.

Predicted TFs	log2FC	P-value	Adjust P-value
SRF	-0.086314752	0.669795783	0.746271948
ELK1	1.558106029	5.56744E-06	2.57606E-05
USF2	0.881276552	2.72281E-19	8.19352E-18
FOXC1	2.873387579	2.54148E-34	3.16654E-32
HNF4A	-3.184609633	1.15639E-05	5.03241E-05
ESR1	-3.572441637	3.80539E-17	8.91416E-16
YY1	-0.088999128	0.174544258	0.252758505
BRCA1	-1.398069107	0.000124605	0.000438504
NFYA	-0.684743419	1.52561E-16	3.33309E-15
TP53	-0.530567058	2.54402E-05	0.000103238
E2F1	-1.803276457	0.000350207	0.001114878
GATA2	-2.263877913	3.21069E-11	3.49238E-10
NRF1	0.079917584	0.422433447	0.519588002
MEF2A	0.356791285	0.093477369	0.149636894

Notes. EMs, endometriosis; NM, normal endometrium; EU, eutopic endometrium; EC, ectopic endometrium (lesions); TF, transcription factor;
